# Imidazole Hybrids: A Privileged Class of Heterocycles in Medicinal Chemistry with New Insights into Anticancer Activity

**DOI:** 10.3390/molecules30102245

**Published:** 2025-05-21

**Authors:** Zarifa Murtazaeva, Azizbek Nasrullaev, Anvarjon Buronov, Shukhrat Gaybullaev, Lifei Nie, Sodik Numonov, Zohidjon Khushnazarov, Davron Turgunov, Rustamkhon Kuryazov, Jiangyu Zhao, Khurshed Bozorov

**Affiliations:** 1 Department of Organic Synthesis and Bioorganic Chemistry, Institute of Biochemistry, Samarkand State University, University Blvd. 15, Samarkand 140104, Uzbekistan; zarifamurtazayeva1992@gmail.com (Z.M.); rustamxonkuryazov@gmail.com (R.K.); 2 State Key Laboratory Basis of Xinjiang Indigenous Medicinal Plants Resource Utilization, Xinjiang Technical Institute of Physics and Chemistry, Chinese Academy of Sciences, South Beijing Rd 40-1, Urumqi 830011, China; nielf@ms.xjb.ac.cn (L.N.); sodikjon82@gmail.com (S.N.); 3 Research Institution “Chinese-Tajik Innovation Center for Natural Products”, National Academy of Sciences of Tajikistan, Dushanbe 734063, Tajikistan; 4 Department of Chemistry, Urgench State University, Kh. Olimjon st. 14, Urgench 220100, Uzbekistan

**Keywords:** anticancer activity, benzimidazole, hybrid compounds, imidazole, imidazole–metal complex, lead compounds, medicinal chemistry

## Abstract

Imidazole is a five-membered heterocyclic system featuring two nitrogen heteroatoms at the 1- and 3-positions of the ring. The imidazole scaffold is particularly suited for kinase inhibition concepts. This further confirms that this scaffold is a privileged structure in the development of anticancer drugs. Considering these key factors and the recent focus of scientists on imidazole compounds, we discuss the anticancer activities of imidazole-containing hybrids and related compounds, highlighting articles published in 2023 that serve as a basis for medicinal chemistry leads. From a chemical perspective, the present review emphasizes hybrid molecules with an imidazole ring in the side chain, imidazole-centered hybrid molecules, condensed imidazole hybrids, hybrid compounds containing two or more imidazole rings, polycyclic imidazole hybrids, imidazole-containing metal complexes, and benzimidazole hybrids.

## 1. Introduction

It is generally understood that the excessive spread of cancer ultimately leads to an increase in the lethality of mankind [1]. Medicinal chemists discovered several “hit” or “lead” compounds to solve this task [2,3,4]. For two cancer treatment modalities, i.e., chemotherapy and immunotherapy, synthesized or natural compounds are used [5,6], especially nitrogen-containing heterocyclic compounds (*N*-heterocycles) [7,8]. Recent reports confirmed the promising anticancer properties of *N*-heterocyclic derivatives [9]. Our research group also investigated the anticancer and other pharmacological potentials of *N*-heterocycles [10,11,12,13,14,15,16,17,18,19,20,21,22,23,24,25,26,27]. On the other hand, several factors (e.g., pharmacokinetics and side effects) become a barrier to the completion of a candidate drug-like agent. Nevertheless, heterocycles are a primary component of chemotherapeutic drugs, and the further development of these compounds seems to be promising.

**Figure 1:**
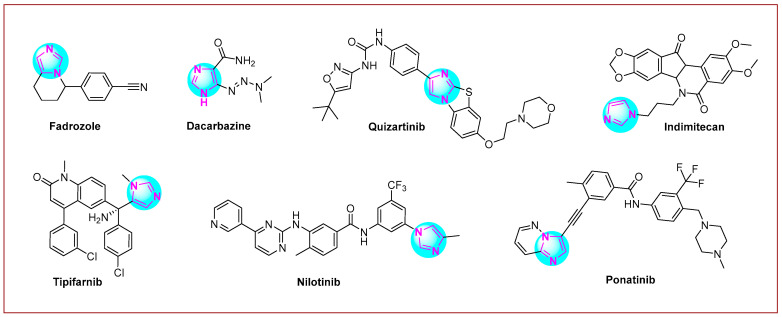
Imidazole-based anticancer drugs are currently undergoing clinical trials.

Imidazole is a five-membered heterocyclic system that contains two nitrogen heteroatoms at the 1 and 3 positions of the ring [28,29,30]. Several compounds with imidazole rings have been found in plants [31] and other living organisms [32]. In addition, these scaffolds are included in many nucleotides; for example, half of the purine structure represents an imidazole ring [33,34]. The imidazole scaffold is more suitable for the concept of kinase inhibition [35]. This again confirms that this scaffold may be a privileged structure for anticancer drug development [36,37,38,39,40]. Many imidazole hybrid drugs are already in use for cancer treatment (Figure 1). **Fadrozole** [41] is a selective non-steroidal aromatase inhibitor used for breast cancer treatment in Japan. Imidazole carboxamide (**dacarbazine** [42]) is also a well-known chemotherapeutic drug used for the treatment of melanoma and Hodgkin’s lymphoma. Another anticancer drug used to treat acute myeloid leukemia is **quizartinib** [43]. **Indimitecan** [44] is a DNA topoisomerase I inhibitor with anticancer activity in several human cancer cell lines. **Tipifarnib** (Zarnestra™) [45] is a farnesyltransferase inhibitor used to treat several types of cancer, including neck cancer, breast cancer, peripheral T-cell lymphoma, and chronic myelomonocytic leukemia. Chronic myeloid leukemia is treated with **nilotinib** (Tasigna^®^) [46], while **ponatinib** [47] is used to treat chronic myeloid leukemia and acute lymphoblastic leukemia.

A “hybrid molecule” integrates two or more pharmacophore substituents, structures, rings, or fragments into a single molecular entity. It may also involve replacing similar components of one compound with those of another. In medicinal chemistry, hybrid molecules can target the same tumor by combining pharmacophore groups from different drugs acting by the exact mechanism [48,49]. Alternatively, they can be designed to target multiple tumors simultaneously by incorporating pharmacophores from medicines that use various mechanisms of action [49]. The synthesis of azole-containing hybrids is easily achieved by CH functionalization of two necessary heterocyclic skeletons [9,50,51,52,53,54,55].

Considering the recent focus on imidazole compounds, we discuss and highlight the anticancer activities of imidazole-containing hybrids and related compounds, based on articles published in 2023, as a basis for medicinal chemistry leads.

## 2. The Literature Acquisition Strategy of This Review

The imidazole core structure has emerged as a significant building block in various drug candidates. Every year, multiple reports have been published that examine their physical and chemical properties. Since 2023, approximately 35 review articles with the keyword “imidazole” in their titles have been published in the Scopus database [56]. However, a majority of these papers focus on the diverse chemical and physical properties of this heterocyclic system. Several review articles have examined the medicinal and pharmacological properties of imidazole hybrids, but more than 100 scientific studies on this topic were published each year. On 13 January 2023, two review articles on the biological properties of imidazole and its derivatives were published by Tolomeu and Fraga [57] and Rani et al. [58]. These publications refer to literature before 2023. In 2024, Poyraz et al. [59] published a comprehensive review of the pharmacological aspects of imidazole hybrids, emphasizing various bioassays. Since 2023, two reviews have addressed the anticancer potential of imidazole-based hybrids: the reviews by Kumar et al. [60] in 2024 and Ghara et al. [61] in 2025. However, Kumar et al. [60] did not include reports published in 2023, while the review by Ghara et al. [61] references only one such report. Our review summarizes, discusses, and highlights imidazole-containing hybrid leads identified as promising drug candidates with anticancer properties based on research investigations reported in 2023.

We searched the Scopus database using the keyword “imidazole” and its variations “imidazole hybrids” and “imidazole AND anticancer” to uncover published reports on the medicinal properties of imidazole and its derivatives, especially for anticancer applications.

We included only reports from 2023 to ensure that our findings reflect the latest research. Studies in medicinal chemistry were prioritized for their relevance to drug development; reports focusing solely on synthetic methods or chemical properties without a pharmacological context were excluded.

We reviewed sources that reported on “imidazole hybrids” identifying lead compounds with anticancer potential, capturing a range of hybrid structures:

(a) Hybrid molecules with an imidazole ring in the side chain: Compounds where the imidazole moiety is part of a larger structure that influences biological activity.
(b) Imidazole-centered hybrid molecules: Compounds where the imidazole serves as a central core interacting with multiple biological targets.
(c) Condensed imidazole hybrids: Structures with fused imidazole rings to enhance pharmacological properties.
(d) Hybrid compounds with two or more imidazole rings: These have unique mechanisms of action and improved efficacy due to multiple imidazole units.
(e) Polycyclic imidazole hybrids: Complex structures incorporating imidazole in a polycyclic framework, affecting solubility and biological interactions.
(f) Imidazole-containing metal complexes: Reports included due to their interesting biological activities from the involved metal ions.
(g) Benzimidazole hybrids: These compounds demonstrate structural variations around the imidazole ring to enhance anticancer activity.

Thus, our search rigorously filtered the literature to include only reports relevant to the medicinal chemistry of imidazole hybrids with documented anticancer activity, allowing for focused analysis of their therapeutic promise.

## 3. Hybrid Molecules with an Imidazole Ring in the Side Chain

**Figure 2:**
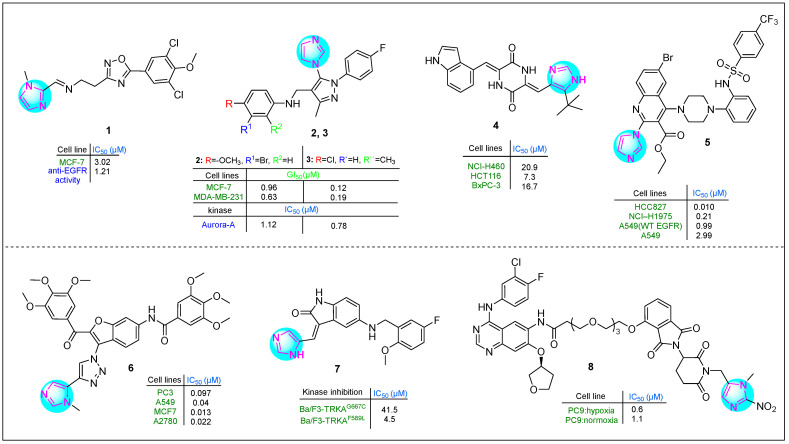
The structures of potential antitumor compounds **1**–**8**.

Several imidazole-1,2,4-oxadiazole hybrids were synthesized and evaluated for their antiproliferative activity by Lavunuri et al. [62]. Erlotinib was used as a reference drug. Most prepared imidazole derivatives exhibited higher potency in MCF-7 breast cancer cells than in A549 lung cancer and HepG2 hepatocellular carcinoma cells. Among them, derivative **1**, namely (*E*)-N-(2-(5-(3,5-dichloro-4-methoxyphenyl)-1,2,4-oxadiazol-3-yl)ethyl)-1-(1-methyl-1H-imidazol-2-yl)mathen-amine (Figure 2), showed better activity against MCF-7 cells (IC_50_: 3.02 µM). This imidazole compound was also promising on HepG2 and A549 cell lines. Compound **1** showed promising anti-EGFR activity, with an IC_50_ of 1.21 µM.

Yevale et al. [63] investigated the anticancer properties of imidazole–pyrazole hybrids. All hybrid compounds were tested against human MCF-7 and MDA-MB-231 breast cancer cells and Vero kidney epithelial cells. Two imidazole compounds, **2** and **3** (Figure 2), containing various substituents on the benzene ring—including halogens and methoxy and methyl groups—exhibited potent cytotoxicity against the MDA-MB-231 (GI_50_: 0.63 μM) and MCF-7 (GI_50_: 12 μM) cell lines. In comparison, imatinib showed GI_50_ values of 10.36 μM for MDA-MB-231 cells and 16.08 μM for MCF-7 cells. Notably, **2** and **3** were more potent inhibitors of Aurora A kinase than alisertib, with **2** being 4.7 times stronger and **3** being 2.8 times more potent. In addition, **2** was effective in arresting the cell cycle at the G2/M phase (34.9%).

Fang et al. [64] studied the antitumor properties of novel plinabulin scaffolds against human H460, HCT116, and BxPC-3 cancer cell lines, focusing on targeting β-tubulin. Derivative **4** (Figure 2) was identified as the most potent compound, with IC_50_ values of 20.9 μM for NCI-H460 cells, 7.3 μM for HCT116 cells, and 16.7 μM for BxPC-3 cells. This hybrid derivative was particularly effective in inhibiting microtubule formation. In addition, it disrupted the proliferation of the HCT116 cell line during the G2/M phase at a concentration of 20 nM.

Quinoline–imidazole–piperidine hybrids, including substituted amide and sulfonamide fragments, were synthesized by Kardile et al. [65]. The compounds were tested against various cancer cell lines, including HCC827 (EGFR Del E746-A750), NCI-H1975 (L858R/T790M), and A549 (wild-type EGFR), as well as normal BEAS-2B bronchial epithelium cells. Imidazole **5** (Figure 2) showed the highest potency, with IC_50_ values of 0.010, 0.21, 0.99, 2.9, 9, and 85.14 μM, respectively. Additionally, it exhibited promising enzyme inhibitory activity against EGFR L858R/T790M in vitro (IC_50_: 138 nM).

Gariganti et al. synthesized interesting hybrid derivatives containing an imidazole fragment [66]. The compounds were tested in PC3, A549, MCF7, and A2780 cell lines using the MTT assay. Derivative **6** (Figure 2), which contains two 3,4,5-trimethoxyphenyl rings in its side chains, a benzofuran nucleus, an amide group, and both 1,2,3-triazole and imidazole moieties, exhibited excellent apoptotic activity. The IC_50_ values for compound **6** in the four cell lines investigated were 0.097, 0.04, 0.013, and 0.022 µM, respectively. This indicates that **6** was 25-, 77-, 162-, and 63-fold more active than the reference drug etoposide.

Qin and colleagues reported several indole derivatives containing an imidazole ring that served as potent and orally bioavailable TRKA inhibitors [67]. Compound **7** (Figure 2) emerged as the lead molecule, demonstrating excellent stability in plasma (half-life > 289.1 min) and moderate stability in liver microsomes (half-life: 44.3 min). Pharmacokinetic studies indicated that compound **7** has good oral bioavailability as a TRKA inhibitor, with a bioavailability rate of 116.07%. In the Ba/F3-TRKA^F589L^ cell line, hybrid **7** showed an IC_50_ of 41.5 µM, while it showed an IC_50_ of 4.5 µM in Ba/F3-TRKA^G667C^ cells. These results indicate that imidazole **7** had superior antiproliferative activity compared to selitrectinib, which had an IC_50_ of 95.6 µM in Ba/F3-TRKA^F589L^ cells and 16.7 µM in Ba/F3-TRKA^G667C^ cells.

Cheng et al. [68] developed a novel proteolysis-targeting chimera (PROTAC) by incorporating a 1-methyl-2-nitro-1*H*-imidazol-5-yl methyl group into an EGFR-based PROTAC containing a CRBN E3 ligand. The inhibitory activity of the target compounds was evaluated in vitro against human PC9 lung adenocarcinoma cells. In particular, imidazole compound **8** (Figure 2) showed significant hypoxia-selective inhibitory activity.

**Figure 3:**
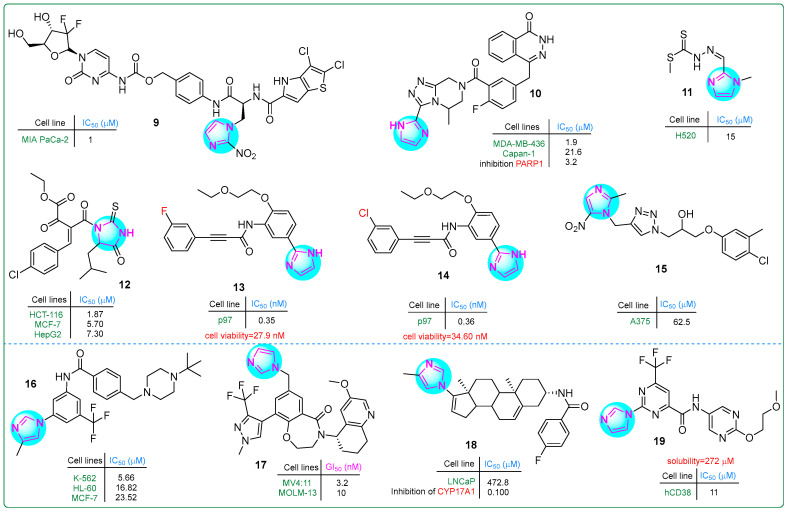
The structures of potential antitumor compounds **9**–**19**.

Tsuji and colleagues [69] developed a dual prodrug improving pancreatic cancer treatment by releasing a PYG inhibitor and gemcitabine under hypoxic conditions. Hybrid compound **9** (Figure 3), which contains an imidazole-(4-aminophenyl)methylcarbamate fragment, exhibited significant antiproliferative effects in a dose-dependent manner at concentrations ranging from 1 to 10 μM.

A series of imidazole derivatives linked by a triazolopyrazine ring were developed as PARP1 inhibitors by Wang et al. [70]. In particular, compound **10** (Figure 3) exhibited exceptional activity (IC_50_: 1.9 ± 0.5 nM), making it 23 times more potent than olaparib (IC_50_: 43.2 nM) against the MDA-MB-436 (BRCA1^−/−^) breast cancer cell line. In addition, it demonstrated an IC_50_ of 21.6 ± 13.7 nM against the Capan-1 pancreatic cancer cell line, which was 32 times more potent than olaparib (IC_50_: 692 nM). Even more significantly, compound **10** inhibited PARP1 activity at an impressively low IC_50_ value of only 3.2 nM.

Malakar and co-workers investigated dithiocarbazate-based Schiff bases containing an imidazole ring [71]. The chemotherapeutic ligand **11** (Figure 3), with IC_50_ values ranging from 70.4 to 2.34 μM, exhibited significant cytotoxicity against the human H520 lung squamous cell carcinoma cell line using the MTT assay. In particular, the presence of amino and thione groups in the Schiff base structure enhanced its antiproliferative activity. This highlights the importance of these features for improved therapeutic efficacy.

Hassan et al. synthesized thioxoimidazolidinone compounds as dual inhibitors of CDK2 and EGFR, with derivative **12** (Figure 3) showing exceptional potency [72]. It achieved IC_50_ values of 0.098 μM for EGFR and 0.087 μM for CDK2. In addition, compound **12** effectively inhibited the growth of HCT-116, MCF-7, and HepG-2 cancer cells, with IC_50_ values of 1.87 μM, 5.70 μM, and 7.30 μM, respectively. In comparison, erlotinib used as a standard drug showed higher IC_50_ values (17.86 μM for HCT116, 13.0 μM for MCF-7, and 72.3 μM for HepG-2 cells).

p97 (valosin-containing protein) is considered a promising target for anticancer therapies. Its expression is elevated in various cancers, including colorectal, pancreatic, thyroid, squamous cell, breast, osteosarcoma, gastric, and lung cancer [73]. Novel hybrid imidazole derivatives were synthesized as covalent inhibitors of p97/VCP ATPase, a protein involved in various cellular processes, by Wang et al. [74]. Derivatives **13** and **14** (Figure 3) exhibited impressive activity, with IC_50_ values of 0.35 nM and 0.36 nM, respectively, against p97. Additionally, cell viability was assessed in the human U87MG glioblastoma cell line for compound **13** (IC_50_: 27.9 nM) and for compound **14** (IC_50_: 34.6 nM). These results may position both compounds as promising candidates for cancer therapy.

In a comprehensive study by Zarenezhad et al. [75], several compounds with imidazole and triazole rings were investigated. Their in vitro cytotoxic activity was tested against the human melanoma A375 cell line using the MTT assay. Compound **15** (Figure 3) exhibited moderate activity; however, structural modifications are necessary for future studies to enhance its potency. Docking analysis revealed that **15** formed multiple hydrogen bonds and hydrophobic interactions in the active regions of the tubulin dimer.

The development of novel BCR-ABL tyrosine kinase inhibitors relevant to chronic myeloid leukemia was reported by Wang and colleagues [76]. They synthesized imidazole-containing aromatic amides through nucleophilic reactions with piperazines and morpholines. These compounds were tested against three human cancer cell lines: K-562 and HL-60 leukemia cells and MCF-7 breast cancer cells. In particular, the imidazole derivative **16** (Figure 3) showed the lowest IC_50_ value against K-562 cells (5.66 ± 2.06 μM). The K-562 leukemia cells were inhibited at lower concentrations than the MCF7 cells. This ensures the specificity of compound **16**, which was further subjected to the other biological assays.

By creating hybrids of benzoxazepinone and imidazole, Teuscher et al. [77] developed novel inhibitors targeting the WDR5 protein. The 3,4-dihydrobenzo [f] [1,4] oxazepin-5(2*H*)-one core structure was an effective scaffold for these inhibitors. In particular, compound **17** (Figure 3) exhibited the strongest growth inhibition (GI_50_), which was as low as 3.2 nM against the MV4:11 myelomonocytic leukemia cell line and 10 nM against the MOLM-13 acute myeloid leukemia cell line.

The hybrid derivative **18** (Figure 3), which combines imidazole and phenanthrene rings, was a promising candidate for prostate cancer therapy [78]. In LNCaP cells, it exhibited significant androgen receptor (AR) degradation activity, with a DC_50_ of 1.28 µM, making it 11.2 times more potent than galeterone (DC_50_: 14.4 µM). In addition, in vitro screening showed that the derivative **18** had an IC_50_ of more than 40 µM, indicating lower hERG channel suppression compared to galeterone, which had an IC_50_ of 6.47 µM. This suggests that **18** may provide effective AR targeting with a potentially better safety profile concerning cardiac effects.

Compound **19** (Figure 3), a lead candidate designed as a CD38 inhibitor, featured bioisosteric structures that include an imidazole moiety on the right, a 2-methoxyethoxypyrimidine ring on the left, and a central 4-fluoromethylpyrimidine. It was synthesized by Li et al. and had an IC_50_ value of 11 μM for CD38 [79]. Its structure incorporated a polar group, resulting in an impressive solubility of 272 mM in phosphate-buffered saline (PBS) at pH 7.4, making it an interesting candidate for further biological investigation and potential therapeutic use.

## 4. Imidazole-Centered Hybrid Molecules

**Figure 4:**
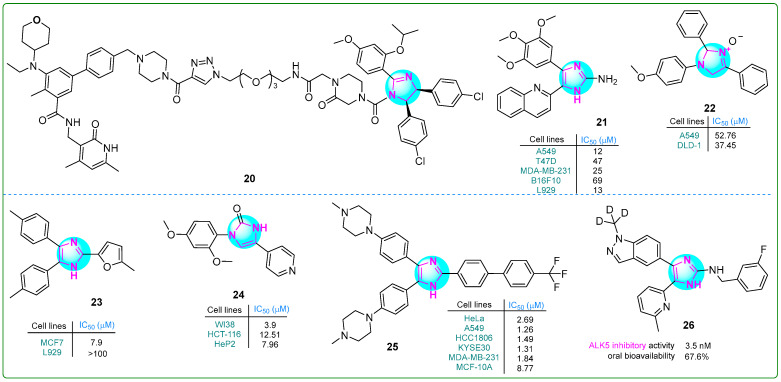
The structures of potential antitumor compounds **20**–**26**.

Xie and colleagues [80] reported the development of a proteolysis-targeting chimera that exhibited six times the anti-cell proliferative activity of the selective EZH2 inhibitor tazemetostat (EPZ-6438; IC_50_: 11 nM). In particular, the compound **20** (Figure 4) showed strong anti-proliferative effects on human Su-DHL-6 large cell lymphoma cells upon treatment with 30 μM for 48 h. In addition, treatment with 10 μM **20** led to significant apoptosis in the same cell line after 48 h.

Giri et al. [81] synthesized compound **21** (Figure 4) with remarkable cytotoxicity against various cancer cell lines. Featuring a 2-aminoimidazole core and a 2,3,4-trimethoxy aromatic ring, compound **21** exhibited potent inhibitory effects on HeLa cervical (IC_50_: 10 nM) cancer cells and B16F10 (47 nM) melanoma cells, as well as T47D (12 nM), MDA-MB-231 (47 nM), and MCF-7 (13 nM) breast cancer cells. The compound effectively disrupted tubulin polymerization, with a dissociation constant of 5.0 ± 0.6 μM. In addition, in silico studies indicated that compound **21** exhibits excellent water solubility and a favorable balance of hydrophilicity and lipophilicity, thereby possibly enhancing its potential for oral administration in cancer treatment.

Muhammed and colleagues developed imidazole-based compounds that exhibit cytotoxic properties against various cancer cells [82]. When the human A549 lung adenocarcinoma and DLD-1 colorectal cell lines were treated with these compounds for 48 h, compound **22** (Figure 4) showed greater efficacy than cisplatin in inhibiting the proliferation. Additionally, molecular docking studies revealed that compound **22** forms a single hydrogen bond with DNA topoisomerase I.

Çetiner et al. investigated hybrid derivatives with imidazole cores, evaluating their antitumor potential against the MCF7 breast cancer cell line using the MTT assay [83]. The imidazole compound **23** (Figure 4) emerged as particularly noteworthy, exhibiting an IC_50_ of 7.9 μM, which is lower than that of most other derivatives and the positive control drug cisplatin (IC_50_: 9.75 μM). However, its anti-aromatase activity (IC_50_: 5.4 μM) did not exceed that of letrozole (IC_50_: 0.114 μM). Furthermore, ligand **23** exhibited the highest interaction energy of −8.224 kcal/mol with the target protein, indicating a strong binding affinity.

Ibrahim and his team reported several imidazole–pyridine hybrids [84]. They tested these compounds for their ability to inhibit the enzyme carbonic anhydrase IX (CA9/CAIX) and their effects on human HCT-116 colon cancer and HeP2 cervical cancer cells. Compound **24** (Figure 4), which has two methoxy groups in the meta position, showed the strongest effects. It had an IC_50_ value of 7.96 μM against HeP2 cells, which was superior to that of doxorubicin as a standard anticancer drug. Compound **24** exhibited the highest toxicity against the HCT-116 cell line (IC_50_: 12.51 μM) compared to other compounds. Additionally, compound **24** significantly inhibited carbonic anhydrase IX (IC_50_: 7.55 μM).

A series of conjugates containing imidazole and various substituted biphenyl fragments were synthesized by Wang et al. [85]. Their antiproliferative effects on human cancer cell lines (HeLa, A549, KYSE30, HCC1806, and MDA-MB-231) were evaluated using the CCK-8 assay. The results indicated that **25** (Figure 4), as the lead compound, exhibited a half-maximum inhibition concentration of 0.66 µM on day 3. Interestingly, this derivative also exhibited low cytotoxicity (IC_50_: 8.77 μM) against normal breast cells, possibly indicating limited tumor-specific effects.

In another study, Kang et al. synthesized a series of deuterated imidazole derivatives as potent ALK5 receptor inhibitors [86]. In particular, hybrid **26** (Figure 4) exhibited high potency in a TGF-β assay (IC_50_: 3.5 ± 0.4 nM) compared to the positive control vacutertib (IC_50_: 9.4 nM). In vivo studies showed that compound **26** had improved microsomal metabolic stability (23.1 μL/min/mg protein) and moderate clearance (29.0 mL/min/kg) in humans.

## 5. Condensed Imidazole Hybrids

**Figure 5:**
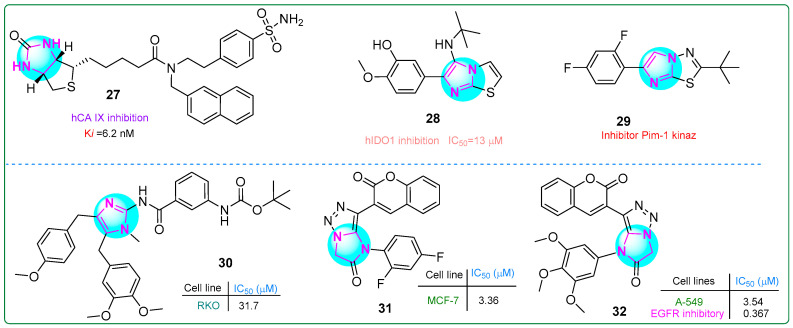
The structures of potential antitumor compounds **27**–**32**.

Begines and colleagues developed a novel hybrid compound that combines an imidazole with a thiophene-condensed sulfonamide group [87]. They investigated its antiproliferative properties by testing it against the cytosolic isoforms hCA I, hCA II, hCA IX, and hCA XII by kinetic analysis. Compound **27** (Figure 5) demonstrated a 61% reduction in proliferation in human PANC-1 pancreatic carcinoma cells. In addition, compound **27** demonstrated the highest potency and selectivity as an inhibitor of human carbonic anhydrase isoform IX (K_i_: 6.2 nM).

Singh et al. [88] synthesized new imidazole derivatives fused to a thiazole ring. These derivatives inhibited aldolase 2,3-dioxygenase 1. Compound **28** (Figure 5) had the lowest half-maximal inhibitory concentration (13 μM) in vitro. Importantly, when tested on, conjugate **28** was non-toxic to cell viability even at a concentration of 100 μM toward human embryonic kidney HEK293 cells, highlighting its safety profile.

Khamees et al. [89] developed new imidazole/thiadiazole derivatives inhibiting the Pim-1 oncoprotein. In silico analysis confirmed that the compound **29** (Figure 5) bound to Pim-1. Furthermore, the drug properties and pharmacokinetic characteristics of **29** were examined using the SwissADME platform, predicting its potential lead candidate for future drug development.

Fu et al. [90] developed analogs of the marine natural product naamidin J that demonstrated antitumor effects on human RKO colorectal adenocarcinoma cells by reducing PD-L1 expression and enhancing tumor-infiltrating T-cell immunity. Compound **30** (Figure 5) exhibited antitumor activity against RKO cells (IC_50_: 31.7 μM). In addition, **30** was identified as an enhancer of intratumoral T-cell infiltration and an inductor of tumor apoptosis.

New bioactive hybrids with anticancer properties, incorporating pharmacophore groups such as imidazole–triazole and coumarin combinations, were developed by Samala et al. [91]. Compound **31** (Figure 5) (IC_50_: 3.36 μM) demonstrated superior activity against MCF-7 breast cancer cells compared to the control drug, erlotinib (IC_50_: 4.25 μM). Additionally, conjugate **32** (Figure 5) was 2.86 times more cytotoxic than erlotinib (IC_50_: 10.12 μM) toward human A549 lung adenocarcinoma cells. Furthermore, compound **32** demonstrated the most potent inhibitory effect on EGFR tyrosine kinase activity (IC_50_: 0.367 µM).

**Figure 6:**
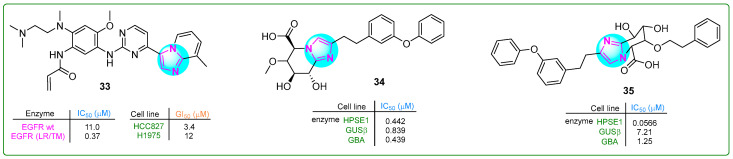
The structures of potential antitumor compounds **33**–**35**.

Liu et al. [92] developed and evaluated a potentially revolutionary new selective and orally bioavailable EGFR YK-029A inhibitor. This inhibitor specifically targeted M and exon 20 insertion mutations and showed promising results in treating non-small cell lung cancer cells. Compound **33** had IC_50_ values of 11.0 nM for the EGFR WT enzyme and 0.37 nM for the EGFR (LR/TM) enzyme. Derivative **33** (Figure 6) is expected to enter Phase III clinical trials for treating EGFRex20 in non-small cell lung cancer.

Compound **34** (Figure 6), a selective inhibitor of heparanase-1 (HPSE-1) that also reduces the inhibitory activity of β-glucuronidase (GUSb) and glucosylceramidase β1 (GBA), was synthesized by Imai and co-workers [93]. Imidazole **34** showed IC_50_ values of 0.442 for heparinase-1 (HPSE-1), while the values for GUSb and GBA were 0.893 and 0.439 μM, respectively. Subsequently, the 6-methoxy group of compound **34** was replaced by a bulky/phenethyloxy fragment, resulting in a new imidazole hybrid **35** (Figure 6) [94]. This hybrid showed enhanced selectivity against HPSE-1 (IC_50_: 0.0566µM) and inhibited the activity of GUSb and GBA by 8.6 and 2.8 times.

**Figure 7:**
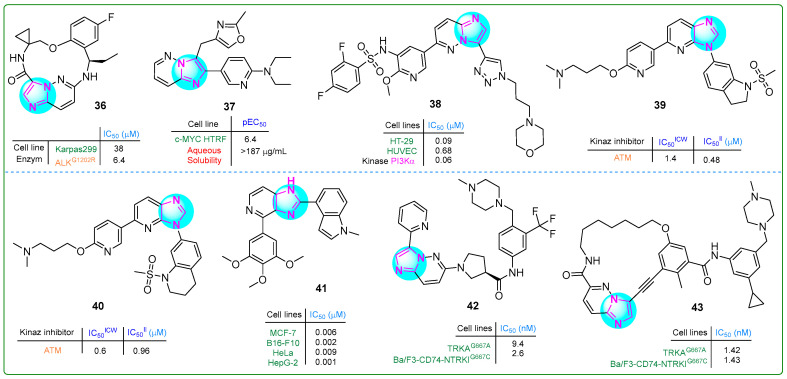
The structures of potential antitumor compounds **36**–**43**.

Novel macrocyclic compounds containing an imidaole moiety were synthesized by Xiao et al. [95] as inhibitors of anaplastic lymphoma kinase (ALK). Their study also evaluated the in vivo pharmacokinetic properties of these compounds. Derivative **36** (Figure 7), which possesses a single chiral atom, exhibited notable superiority in inhibitory activity against the ALKG1202R enzyme (IC_50_: 6.4 μM) compared to reprotectinib, which has two chiral atoms and an IC_50_ value of 7.9 μM.

Marco et al. [96] aimed to improve the pharmacokinetic properties of previously synthesized benzimidazole-based compounds targeting C-MYC by introducing a nitrogen atom into the benzene ring. Their efforts resulted in the creation of a novel biaryl compound, **37** (Figure 7), featuring a 6,5-linked imidazopyridine nucleus. This compound not only demonstrated an improved pEC_50_ value of 6.4 for c-MYC HTRF but also showed improved lipophilicity (5.0) and solubility (≥0.52 µM).

Li et al. [97] developed a series of hybrid compounds featuring an imidazopyridine core, and their anticancer effects were assessed through in vitro and in vivo investigations. Derivative **38** (Figure 7) emerged as a potent inhibitor of mTOR and all classes of PI3K, exhibiting superior activity compared to copanlisib. Furthermore, **38** demonstrated an IC_50_ value of 0.09 μM and 0.07 μM against the HT-29 colon carcinoma and PC-3 prostate carcinoma cell lines, respectively.

Dimitrov et al. studied novel sulfonamide-based ATM kinase inhibitors featuring an imidazopyridine core [98]. Hybrids **39** and **40** (Figure 7), exhibiting the highest nanomolar efficacy, were identified as potential candidates (IC_50_ values of 0.48 and 0.96 μM, respectively).

Ren et al. [99] synthesized new imidazole analogs targeting tubulin polymerization. They tested the compounds in vitro on four cancer cell lines, i.e., MCF-7, B16-F10, HeLa, and HepG-2. Compound **41** (Figure 7) showed more potent antiproliferative activity in all cell lines tested than the positive control, colchicine. The IC_50_ values of this compound ranged from 0.009 to 0.001 nM.

A series of novel derivatives of 6-(pyrrolidine-1-yl)imidazo [1,2-b]pyridazine were synthesized and evaluated for their potential as TRK type II inhibitors by Xiang et al. [100]. Compound **42** (Figure 7) exhibited 109.4-fold more potent antiproliferative activity than the type I TRK inhibitor larotrectinib (IC_50_: 284.4 nM) and 16-fold more significant activity than the type II TRK inhibitor celitrectinib (IC_50_: 41.4 nM) against murine mutant Ba/F3-CD74-TRKAG667C pro-B cells.

Building on previous research, these scientists [101] designed inhibitors targeting TRK type III. The biochemical kinase assay revealed that compound **43** (Figure 7) inhibited TRKG667C at an impressive sub-nanomolar range of 1.42 nM, outperforming celitrectinib 146.5-fold and repotrectinib 30-fold. In addition, this conjugate was effective against Ba/F3-CD74-NTRKTG667C cells, also at a sub-nanomolar level of 1.43. These results demonstrate that compound **43** was superior to other type II TRK inhibitors.

**Figure 8:**
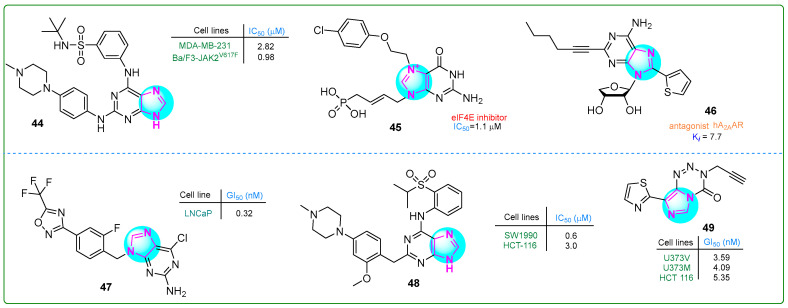
The structures of potential antitumor compounds **44**–**49**.

Guo et al. [102] developed new purine sulfonamides targeting Janus kinase 2 (JAK2) and BRG4 (BD2) with synergistic effects. The enzymatic IC_50_ values for compound **44** (Figure 8) were 22 nM for JAK2 and 13 nM for BRG4. In addition, MV4-11 myelomonocytic leukemia and MDA-MB-230 breast adenocarcinoma cells showed significant inhibitory effects on human cancer cell lines, with IC_50_ values of 1.4 nM and 2.82 nM, respectively. Additionally, a positive therapeutic impact against myeloproliferative disease was noted in the Ba/F3-JAK2V617F allograft model treated with **44** at a dose of 60 mg/kg for 17 days, resulting in normalization of liver and spleen volumes by 44.67% and 59.3%, respectively.

Cárdenas et al. [103] identified novel cell-permeable inhibitors of the eukaryotic translation initiation factor 4E (eIF4E) to block aberrant cap-dependent translation in cancer. The biochemical inhibitory activity and target interaction were assessed for competitive inhibition of eIF4E using a fluorescence polarization assay with fluorescein-labeled m7GTP. Among the synthesized compounds, compound **45** (Figure 8) was the most potent cap analog (IC_50_: 1.1 μM).

Kim and colleagues [104] developed derivatives of 2,8-disubstituted-6-*N*-substituted-4-thionucleosides as potential immuno-oncological agents. Derivative **46** (Figure 8) demonstrated the most substantial antagonism against hA2AAR, displaying the highest affinity (K_i_, A2A: 7.7 ± 0.5 nM). The in vivo pharmacokinetic assessment of the purine-based compound **46** showed an average plasma clearance of 60.5 mL/min/kg. It was absorbed within 15 min upon oral intake, producing an exposure rate of 22.8%.

Moi et al. described compound **47** (Figure 8), which contained a purine base and a trifluoromethyloxydiazole ring [105]. This compound exerted antitumor activity toward human LNCaP prostate adenocarcinoma cells in vitro. In particular, compound **47** effectively inhibited the invasive behavior of prostate cancer cells at a concentration of 0.32 ± 0.02 nM. In addition, inhibitor **47** reduced HDAC4 activity, which contributes to the aggressive cancer characteristics.

Chen and colleagues [106] synthesized novel purine-based imidazole derivatives as DCLK1 inhibitors for treating pancreatic cancer. Compound **48** (Figure 8) significantly inhibited the human SW1990 pancreatic adenocarcinoma cell line at a concentration of 0.6 μM over 21 days. It induced G0/G1 cell cycle arrest and apoptosis.

Summers et al. [107] synthesized thiazole derivatives of imidazotetrazinones with the aim to generate modified substances of temozolomide, a clinically established DNA methylating anticancer drug. Analog **49** (Figure 8) exhibited cytotoxicity against human U373V and U373M astrocytoma cells as well as HCT 116 colon carcinoma cells in vitro at concentrations of 3.59, 4.09, and 5.35, respectively. This compound demonstrated a far greater selectivity than temozolomide.

## 6. Hybrid Compounds Containing Two or More Imidazole Rings

**Figure 9:**
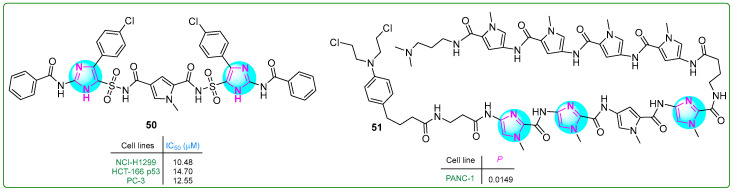
The structures of antitumor compounds containing two or more imidazole rings **50** and **51**.

Sowmya and her team [108] developed a new series of bis-imidazosulfonylpyrrolidicaboxamides and evaluated their cytotoxic effects on NCI-H1299 non-small cell lung cancer, HCT-166 colon cancer, and PC-3 prostate cancer cell lines. Compound **50** (Figure 9) exhibited the most potent cytotoxic effects, with IC_50_ values of 10.48, 14.7, and 12.55 nM after treatment for 24 h. Furthermore, molecular docking revealed that **50** exhibits the highest protein binding energy (∆Gb: −12.86 kcal/mol) and the lowest predicted inhibition constant (pK_i_: 0.38 nM).

Hirose and colleagues [109] developed pyrrole–imidazole polyamide analogs, a class of hybrid compounds that act as DNA alkylating agents. The authors tested them in p53-mutated PANC-1 prostate xenograft tumors. The intravenous administration of compound **51** (Figure 9) (1 mg/kg) along with gemcitabine to mice for three weeks inhibited tumor growth 50 times more than gemcitabine (50 mg/kg) alone, demonstrating the potential of pyrrole–imidazole polyamide analogs in cancer treatment.

**Figure 10:**
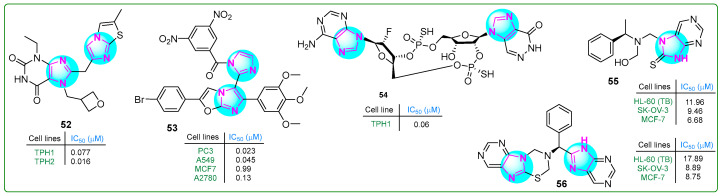
The structure of antitumor compounds with two or more condensed imidazole rings **52**–**56**.

Specker et al. [110] developed tryptophan hydroxylase inhibitors in order to suppress serotonin overproduction. Imidazole compound **52** (Figure 10), a xanthine derivative of imidazopyridine–imidazothiazoles, exhibited the highest inhibitory potency and microsomal stability. Hybrid **52** inhibited the TPH1 and TPH2 isoforms at sub-nanomolar concentrations of 0.007 and 0.016 μM, respectively, with a solubility coefficient of 189 μM.

Syed et al. [111] synthesized imidazole–oxazole derivatives with two imidazole rings and evaluated their activity against PC3 prostate cancer, MCF7 breast cancer, A549 lung adenocarcinoma, and A2780 ovarian carcinoma cell lines using the MTT assay. Analog **53** (Figure 10) exhibited the most significant anticancer properties. Additional structure–activity relationship studies indicated that compound **53** contained an electron-withdrawing (3,5-dinitro) substituent on the phenyl group, resulting in the highest anticancer activity (PC3, IC_50_: 0.023 μM; A549, IC_50_: 0.045 μM; MCF-7, IC_50_: 0.99 μM; and A2780, IC_50_: 0.13 μM). These results suggest that compound **53** was significantly more potent than etoposide.

Kuttruff et al. [112] developed a novel selective imidazopyridazinone-β-D-ribofuranoside-(2′→5′)-phosphorothioate-2′-F-2′-deoxyadenosine-(3′→5′)-phosphorothioate (compound **54**, (Figure 10)) STING agonist. This cyclic dinucleotide (**54**) activated all five STING variants (WT, REF, HAQ, AQ, and Q alleles) at low IC_50_ concentrations (0.54, 0.64, 6.11, 0.61, and 1.98 μM, respectively). The effectiveness of the STING agonist **54** against tumors was assessed in vivo utilizing the murine EMT6 syngeneic breast cancer model. Doses of 0.25, 1, or 4 μg were given weekly for three weeks, leading to total regression of tumor cells even at the lowest dose.

Hassan et al. [113] presented new analogs of chiral imidazole compounds with a condensed bi-tri-heterocyclic structure. These compounds were synthesized using the Mannich reaction and evaluated for cytotoxic activity against 60 cancer cell lines at the US National Cancer Institute. The biological screening revealed that two hybrids, **55** and **56**, (Figure 10) exhibited significant activity against various cancer cell types at a single concentration of 10^−5^ M. Specifically, the analog **55** inhibited five leukemia cell lines (73.57% to 82.55%), as well as colon (58.89–80.81%), prostate (57.62–66.72%), and ovarian (54.55–75.25%) cancer cell lines. Notably, **55** killed 89.94% of LOX IMVI melanoma cells and inhibited the breast cancer cell lines MCF7 and MDA-MB-468 by 94.62% and 99.76%, respectively. Similarly, conjugate **56** showed inhibitory effects on numerous cancer cell types. In particular, it inhibited MDA-MB-468 cells by 92.64% at 10^−5^ M and exhibited 73.68% anti-growth activity against HOP-62 non-small cell lung cancer cells. Furthermore, HL-60 leukemia, MCF7 breast cancer, and SK-OV-3 ovarian cancer cells, as well as VERO normal kidney epithelial cells, were used to test the in vitro cytotoxicity of derivatives **55** and **56**. Compound **55** showed an efficacy comparable to the standard anticancer drug doxorubicin and was 1.66 times more effective than 5-fluorouracil against HL-60 cells. In addition, **55** and **56** showed a 2.5-fold and 2-fold improvement in antiproliferative activity, respectively, compared to 5-fluorouracil in MCF-7 cells.

## 7. Polycyclic Imidazole Hybrids

**Figure 11:**
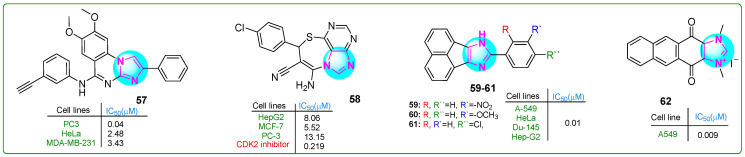
The structure of polycyclic imidazole derivatives **57**–**62** exhibits antitumor activity.

Hasanvand et al. [114] designed new imidazoquinazoline derivatives as tyrosine protein kinase inhibitors, particularly the epidermal growth factor receptor (EGFR). Kinase assay results indicated that compound **57** (Figure 11) showed strong inhibitory potency and selectivity against EGFR (IC_50_: 12.3 μM) among the synthesized compounds. Furthermore, compound **57** showed significant antiproliferative effects against PC3 prostate carcinoma, HepG2 hepatocellular carcinoma, HeLa cervical cancer, and MDA-MB-231 breast cancer cell lines, with IC_50_ values of 0.04, 18.86, 2.48, and 3.43 μM, respectively. Overall, **57** proved to be an exceptional anticancer agent with remarkable antiproliferative activity (PC3, IC_50_: 0.04 μM; HeLa, IC_50_: 2.48 μM; MDA-MB-231, IC_50_: 3.43 μM) that was 145.75, 3.52, and 2 times more effective than erlotinib.

Husseiny et al. [115] prepared the imidazole hybrid **58** (Figure 11) with an imidazolthiazepine moiety, which showed improved antiproliferative effects against the MCF-7 breast cancer and HepG2 hepatocellular carcinoma cell lines compared to the reference medication roscovitine. This purine compound combined showed anticancer activity in the sub-nanomolar range, with IC_50_ values of 8.06 and 5.52 µM for HepG2 and MCF-7 cells, respectively. It exhibited lower cytotoxicity in normal WI38 lung fibroblasts (IC_50_: 39.46 µM). In addition, analog **58** inhibited CDK with an IC_50_ of 0.219 nM, and treatment with this derivative resulted in 58 times more apoptotic cells than the standard treatment.

Sadula and Gaddhe [116] synthesized acenaphthoxynon–imidazole derivatives and performed in silico molecular docking and MTT assays. Compounds **59**–**61** (Figure 11) exhibited higher binding affinities with heat shock protein 90 and DNA topoisomerase II (−10.6; −10.9; −9.3 kcal/mol, respectively) compared to doxorubicin (−9.1 kcal/mol). The effectiveness of these inhibitors was assessed against A549 lung adenocarcinoma, HeLa cervical cancer, Du-145 prostate cancer, and Hep-G2 hepatocellular carcinoma cell lines using the MTT assay. Compounds **59**–**61** exhibited six times greater efficacy than the positive control doxorubicin. These analogues exhibited low toxicity when tested against NHDF normal dermal fibroblasts.

Yuan and colleagues [117] developed novel polycyclic imidazole compounds. Analog **62** (Figure 11) effectively inhibited the growth of A549 lung adenocarcinoma cells (IC_50_: 9 nM). It showed more excellent selectivity, since normal L929 cells were inhibited at much higher concentrations (IC_50_: 21.5 μM)—a result that was even better than those with the first inhibitor YM155. Compound **62** arrested the cell cycle in the G0/G1 phase in the 0–0.1 μM concentration range, with 92.37% of the cells undergoing apoptosis at a dose of 0.25 μM. In addition, in vivo analyses suggested that **62** was more effective than YM155 and doxorubicin in a lung cancer xenograft model. It did not induce side effects or weight loss if administered subcutaneously at a dose of 2 μg/kg.

## 8. Imidazole-Containing Metal Complexes

**Figure 12:**
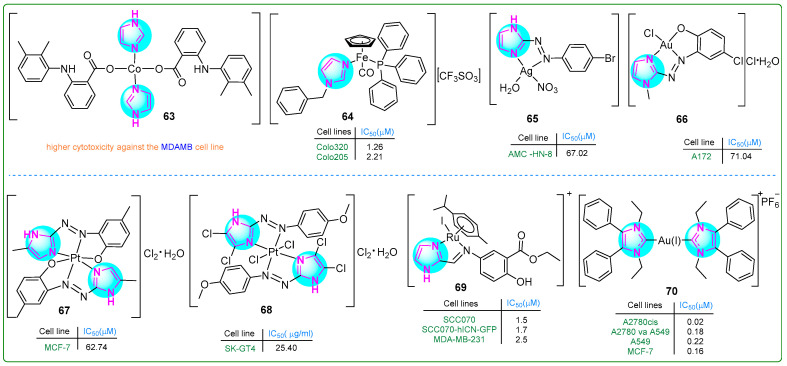
The structures of imidazole complexes (**63**–**70**) exhibit antitumor activities.

Cobalt (Co) metal complexes featuring imidazole and mefenamic acid ligands were synthesized by Nnabuike et al. [118]. The structural configurations of the synthesized complexes were determined using crystal X-ray diffraction analysis. Complex **63** (Figure 12) exhibited significantly stronger anticancer properties than mefenamic acid toward MDA-MB breast cancer cells.

Pilon et al. [119] synthesized new organometallic imidazole compounds containing iron (Fe) and evaluated their efficacy against human Colo320 and Colo205 colon adenocarcinoma cell lines, as well as human MRC-5 embryonic fibroblasts. Treatment with compound **64** (Figure 12) for 48 h resulted in IC_50_ values of 1.26 μM for Colo320, 2.21 μM for Colo205, and 3.1 μM for MRC-5 cells. Compound **64** demonstrated efficacy that was superior to cisplatin and comparable to doxorubicin. In addition, compound **64** inhibited the activity of P-glycoprotein (ABCB1), a key player in multidrug resistance in cancer. It induced both early (27.9%) and late (34.4%) stages of apoptosis in Colo320 cells.

Azal Shakir Waheeb [120] conducted a comprehensive study of imidazole metal complexes chemically linked to aromatic amines through the azo group of the -N = N- chromophore. The silver(I) complex **65** (Figure 12) inhibited the proliferation of primary human laryngeal epithelial cell lines, particularly the AMC-HN8 cell line, which demonstrated a moderate IC_50_ value of 67.02 µM. Importantly, this compound exhibited relatively lower cytotoxicity against WRL-68 normal fetal liver cells (IC_50_: 151.7 µM). These results suggest that complex **65** may exhibit selectivity by being effective against cancer cells but sparing normal cells.

Al-Adilee and colleagues investigated a novel class of metal chelate complexes containing azo-imidazole moieties [121,122,123]. The Au(III) complex **66** (Figure 12) exhibited cytotoxic effects, eliminating cancer cells only at a concentration of 71.04 nM. In contrast, normal WRL-68 cells showed significantly lower sensitivity (IC_50_: 169.6 μM), indicating a relatively low cytotoxic effect on healthy cells [122]. The Pt(IV) complex **67** (Figure 12) exhibited cytotoxic properties against breast cancer cell lines. Quantitatively, the complex induced 53.24% cell death at a concentration of 0.28 µM, with an IC_50_ value of 0.09 µM. While these cytotoxic levels do not exceed those of the standard chemotherapeutic drug doxorubicin, which has an IC_50_ of 0.07 µM, it is noteworthy that the Pt(IV) complex **67** showed an improved safety profile, with an IC_50_ for healthy WRL-68 cells of 0.13 µM [123]. Subsequently, a Pt(IV) complex **68** (Figure 12) was synthesized that exhibited potent inhibitory effects on human esophageal carcinoma cells through a ligand exchange mechanism [121]. In particular, the chloride salt derivative of this complex exhibited promising anticancer activity against the SK-GT4 esophageal carcinoma cell line, demonstrating effectiveness at a nanomolar concentration of 28.32 nM. In addition to its anticancer properties, this novel chelate complex also exhibited significant antioxidant activity, suggesting a broad potential for therapeutic applications.

Simultaneously, Kumari et al. [124] developed 15 new Ru(II) complexes using imidazolyl mesalazine esters. Three of them showed significantly better antiproliferative effects than CDDP as a standard drug. In particular, compound **69** (Figure 12) effectively decreased the c-MYC levels in SCC070 and SCC070-hICN-GFP oral squamous carcinoma cells, as well as MDA-MB-231 breast cancer cells, at concentrations of 1.5, 1.7, and 2.5 nM. Additionally, complex **69** outperformed the other two compounds in promoting mitochondrial depolarization.

Kapitza et al. [125] designed Au(I/III) complexes attached to the imidazole ring and evaluated their biological activity using the MTT assay. Upon incubation with compound **70** (Figure 12) for 72 h, a panel of drug-sensitive and -resistant cancer cell lines (A549, A549-R, K562, K562-R, MCF-7, MCF-7TamR) exhibited IC_50_ values in the range of 0.14 to 0.55 µM, and the drug-resistant sublines did not show cross-resistance to compound **70**. The same was true for another set of sensitive and resistant cell lines (A2780, A2780V-CSC, A2780cis, IGROV1, and IGROV1-CSC) (IC_50_: 0.02 to 1.04 nM).

**Figure 13:**
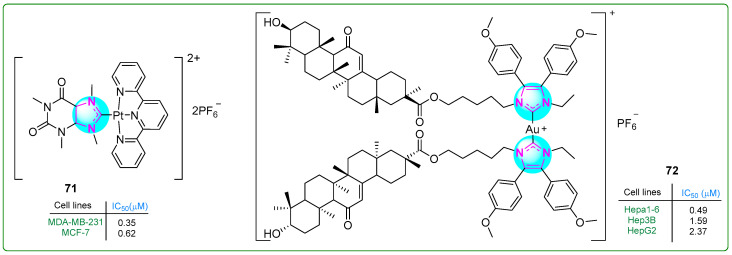
The structures of imidazole complexes **71** and **72**, which exhibit antitumor activities.

Zhang et al. [126] prepared a new platinum(II) complex **71** (Figure 13) with a purine moiety and evaluated it in vivo and in vitro. This complex acted as a multimodal anticancer agent, damaging mitochondria and inhibiting several metabolic pathways often overactivated in triple-negative breast cancer, mainly carbohydrate, lipid, and nucleotide metabolism. In addition, it activated an anticancer mechanism that promotes autophagy, including mitophagy. The novel platinum(II) complex **71** exhibited antiproliferative activity on MDA-MB-231 (IC_50_: 0.35 μM) and MCF-7 breast cancer cell lines (IC_50_: 0.62 μM), demonstrating an antiproliferative effect 60 times more potent than that of cisplatin (IC_50_: 21.0 μM).

Yang et al. [127] studied NHC-Au(I) complexes containing 4,5-diaryl imidazole and glycyrrhetinic acid. Complex **72** (Figure 13) was the most effective one, inducing immunogenic cell death in hepatocellular carcinoma cells. The antiproliferative effects of the complexes were evaluated using the MTT assay on Hepa1-6 (IC_50_: 0.49 μM), Hep3B (IC_50_: 1.59 μM), and HepG2 (IC_50_: 2.37 μM) hepatocellular carcinoma cell lines and normal liver cells (LO2: 3.49 μM). Complex **72** also demonstrated more significant cytotoxicity than auranofin, oxaliplatin, and cisplatin, which were used as positive controls.

## 9. Benzimidazole Hybrids

**Figure 14:**
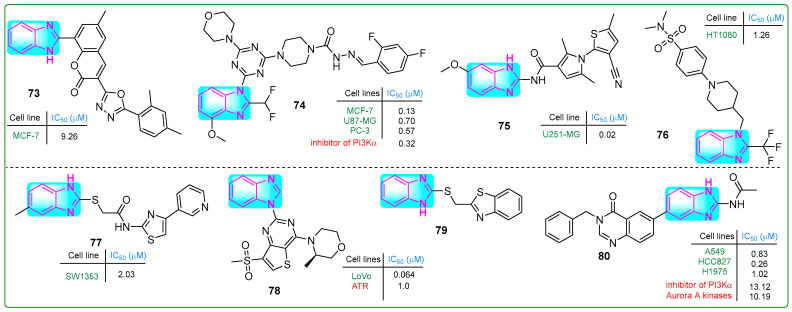
The structure of benzimidazole hybrids (**73**–**80**) shows antitumor activity.

Patagar and colleagues synthesized hybrid **73** (Figure 14), which integrated benzimidazole, oxadiazole, and coumarin nuclei, evaluating its efficacy against MCF-7 breast cancer using MTT assays [128]. Compound **73** exhibited weak anticancer activity (IC_50_: 20.67 µM) comparable to that of the standard drug doxorubicin. In addition, the in silico binding energy of conjugate **73** to quinone reductase was −11.5 kcal/mol, which was considerably lower than that of doxorubicin (−7.9 kcal/mol).

Wang et al. [129] generated new benzimidazole–carbohydrazide hybrids. In the ADP-Glo fluorescent kinase assay, compound **74** (Figure 14) demonstrated PI3Kα enzyme inhibition at a remarkably low concentration of 0.32 nanomoles. Compound **74** was subsequently tested in vitro, demonstrating inhibitory activity toward PC-3 prostate cancer, MCF-7 breast cancer, and U87.MG glioblastoma cells at concentrations of 0.57, 0.13, and 0.7 µM, respectively. These results are comparable to those observed with the reference compound ZSKT-474. Oral and intravenous injections of compound **74** were administered daily to xenograft-transplanted mice for 17 days, resulting in a 57.7% reduction in tumor size.

Recognizing that the DNA-cleaving activity of DHX33 helicase during DNA replication can lead to cancer, Wang and coworkers [130] designed DHX33 small-molecule inhibitors. Carboxamide **75** (Figure 14), which contained pyrrole, thiophene, and benzimidazole structures, was the most potent inhibitor of DHX33. In human liver microsomal stability assays using U251.MG glioblastoma cells, hybrid **75** demonstrated 391-fold higher potency (IC_50_: 0.02 μM) than the previously synthesized BCD38 (IC_50_: 7.82 μM).

Fang et al. [131] developed compound **76** (Figure 14), which inhibited the cystine–glutamate antiporter and induced cell death by ferroptosis. Compound **76** effectively reduced the proliferation of various human cancer cell lines, including 786-O, MDA-MB-231, HeLa, A375, and Du145, with IC_50_ values of 0.7, 4.34, 1.91, 1.33, 2.31, and 1.64 μM, respectively. Since **76** (IC_50_: 15.6 μM) is more metabolically stable than the known ferroptosis inducer erastin (IC_50_: 4.0 μM), further in vivo studies were conducted. Intraperitoneal injection of **76** for 21 days in a HepG2 xenograft tumor model resulted in ferroptosis and a 77.1% reduction in tumor masses without side effects.

Coşkun and colleagues reported a new class of 4-(2/3/4-pyridyl)thiazole-2-acetamides with a benzimidazole side chain [132]. Compound **77** (Figure 14) inhibited the human chondrosarcoma cell line SW1353, with an IC_50_ value of 2.03 μM. In addition, normal L929 connective tissue cells showed low cytotoxicity (IC_50_: 356.73 µM). Conjugate **77** increased the levels of the pro-apoptotic BAX protein while decreasing the levels of the anti-apoptotic BCL-2 protein, thereby inducing apoptosis. It also effectively inhibited tyrosine kinase activity.

Duan et al. [133] developed a series of benzimidazole–thienopyridine analogs that inhibit ATP kinase. Some of these compounds demonstrated better effects than the reference drug, BAY 1895344. Notably, compound **78** (Figure 14) stood out among the others. It exhibited a bioavailability of 32.8%, a short half-life (T1/2: 1.24 h), and a clearance rate of 4495 mL/kg/h. Compound **78** effectively and selectively inhibited ATR kinase at a concentration of 1.0 nM.

Radwan et al. synthesized a hybrid compound **79** (Figure 14) with a unique structure comprising thione-linked benzimidazole and benzothiazole moieties [134]. Conjugate **79** did not exhibit significant antitumor activity toward A549 lung adenocarcinoma cells. Nevertheless, this compound inhibited cell migration at a concentration of 20 μM. It also bound to the heterogeneous nuclear ribonucleoprotein (hnRNP) protein, suggesting possible biological effects.

Fan et al. [135] synthesized a new compound **80** (Figure 14) by adding a benzyl group at the N3 position of the derivative 6-(2-amino-1*H*-benzo[d]imidazol-6-yl)quinazolin-4(3*H*)-one, which inhibited Aurora A and PI3Kα kinase activity, with IC_50_ values of 10.19 and 13.12 nM, respectively. Furthermore, hybrid **80** exhibited significant antitumor activity against three lung cancer cell lines (A549, HCC827, and H1975), with IC_50_ values of 0.83, 0.26, and 1.02 μM, respectively, and it promoted apoptosis. Additionally, conjugate **80** displayed a 7-fold reduction in cytotoxicity (IC_50_: 1.87 μM) against normal human MRC-5 embryonic lung fibroblasts.

**Figure 15:**
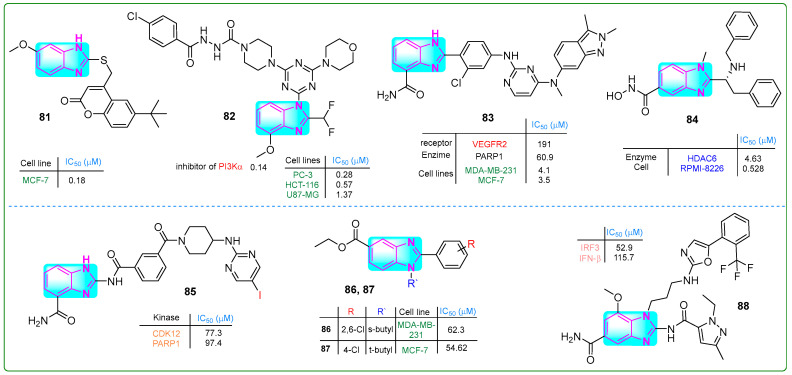
The structures of benzimidazole hybrids (**81**–**88**) with antitumor activity.

Kadam and his research team [136] investigated imidazole hybrids linked to coumarin through a thiol group. They evaluated the cytotoxic effects with paclitaxel as the reference drug. The hybrid **81** (Figure 15) exhibited a superior antiproliferative effect (IC_50_: 0.18 ± 0.13 μM) against MCF-7 breast cancer cells using MTT assays that was better than the effect of paclitaxel (IC_50_: 0.35 μM). In addition, molecular docking studies revealed that compound **81** had a protein binding affinity of −9.2 kcal/mol, which was significantly better than the standard compound isoniazid (−5.9 kcal/mol).

Fu and colleagues [137] developed N’-(4-chlorobenzoyl)-4-(4-(4-(2-(difluoromethyl)-4-methoxy-1*H*-benzo[d]imidazol-1-yl)-6-morpholino-1,3,5-triazin-2-yl)piperazine-1-carbohydrazide (**82**, (Figure 15)), a novel selective PI3Kα inhibitor with an IC_50_ of 0.14. This compound exhibited significant antiproliferative effects against PC-3, HCT-116, and U87.MG cell lines in the nanomolar range (0.28, 0.57, and 1.37 nM, respectively). Hybrid **82** was more cytotoxic than the reference drug ZSTK-474 regarding kinase inhibition against these three cancer cells. Additionally, pharmacological assays indicated that **82** induced apoptosis in U87-MG cells and showed that no weight loss or mortality was observed in mice treated with **82**.

Veliparib contains a benzimidazole fragment, while pazopanib is mainly functionalized by benzopyrazole and pyrimidine moieties. Li et al. [138] contributed to developing the PARP inhibitor veliparib and the VEGFR pazopanib. The combination of these two drugs resulted in the dual inhibitor **83** (Figure 15), which effectively inhibited the enzymatic activities of VEGFR2 and PARP1 at doses of 190.6 nM and 60.9 nM, respectively. Compound **83** successfully reduced the proliferation of breast cancer cell lines (MDA-MB-231, MCF-7, HCC1937, and MDA-MB-436) at concentrations of 4.1, 3.5, 2.7, and 1.9 nM, respectively.

Liu and colleagues [139] presented hybrid **84** (Figure 15), which selectively targeted HDAC6. With a benzimidazole core, this compound suppressed HDAC6 at a concentration of 4.63 µM. In xenograft tumor studies in mice injected with RPMI-8226 plasmocytoma cells, derivative **84** at a dose of 0.5 nM for 21 days resulted in an antiproliferative response. In vivo studies further demonstrated that derivative **84**, with a half-life of 0.699 h, resulted in significantly fewer adverse effects, including weight loss and mortality, compared to ACY-1215.

Triple-negative breast cancer is a highly aggressive form of malignant epithelial tumor originating from mammary tissue. Effective treatment combines synergistic approaches, including CDK12 and PARP1 inhibitors. Derivative **85** (Figure 15), synthesized by Zhang et al. [140], exhibited the most potent inhibitory activities against both CDK12 (IC_50_: 285 nM) and PARP1 (IC_50_: 34 nM) alongside a significant antiproliferative effect in triple-negative breast cancer cell lines (IC_50_: 8.5 μM in MB-231; IC_50_: 9.7 μM in MDA-MB-468; IC_50_: 9.0 μM in -MB-436).

Abdullah et al. [141] developed a new category of 2-*N*-sec-butyl- and tert-butyl-2-arylbenzimidazoles. Compound **86** (Figure 15) inhibited the proliferation of MDA-MB-231 cells at an IC_50_ of 62.3 µM, and other hybrid **87** inhibited the proliferation of MCF-7 breast cancer cells at an IC_50_ of 54.62 µM (Figure 15).

Chen and colleagues [142] presented new derivatives of essential monoaminobenzimidazole compounds. Compound **88** (Figure 15) emerged as a STING agonist among these synthesized compounds due to its favorable pharmacokinetic properties. Specifically, compound **88** activated IRF3 with an IC_50_ value of 0.0529 and demonstrated an IFN-β stimulation value of 0.1157 μM. The metabolic stability assay indicated that the half-life for human liver microsomal stability was 44.42 min, with a clearance rate of 15 μL/min/mg.

**Figure 16:**
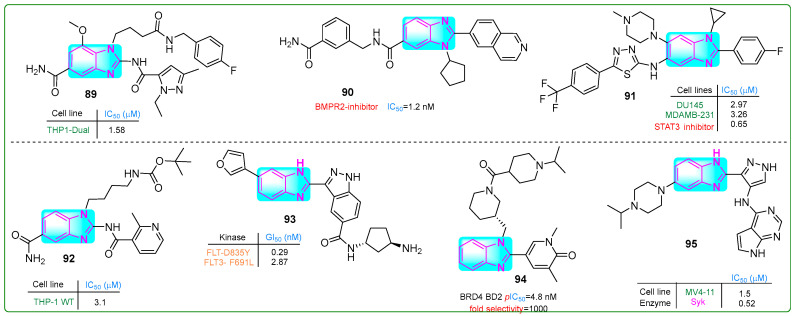
The structures of benzimidazole containing potential antitumor compounds **89**–**95**.

Liu et al. [143] synthesized compound **89** (Figure 16), a robust STING agonist derived from amido benzimidazole with an IC_50_ value of 1.5 μM. After 2 h incubation, its metabolic stability was measured at 39.5%. Administration of doses of 0.27, 0.12, and 0.03 μM significantly increased the protein levels of IFN-β, CXCL10, and IL-6, respectively.

Modukuri et al. [144] synthesized **90** (Figure 16), a compound with an isoquinoline and benzimidazole scaffold, which was identified as a selective inhibitor of monokinase targeting bone morphogenetic protein receptor 2 (BMPR2). At a concentration of 1.2 nM, the conjugate **90** exhibited an IC_50_ for BMPR2 inhibition with a half-life of 45 min in MLM and 80 min in HLM. Additionally, the clearance rate of hybrid **90**, which reflects tissue redistribution, was 31 μL/min/mg in MLM and 17 μL/min/mg in HLM. In addition, compound **90** inhibited BMP-bn-induced transactivation in BMP HEK293T reporter cells exposed to 10.21 µM BMP2 for 6 h, with an IC_50_ of 6.19 μM.

Wang et al. [145] identified potent, selective small molecule STAT3 inhibitors with benzimidazole and 1,3,4-thiadiazole structures. Compound **91** (Figure 16) effectively blocked the IL-6/JAK/STAT3 pathway with an IC_50_ of 0.65 μM. Investigation of the antitumor activity of hybrid compound **91** revealed that its doses for inhibiting proliferation in DU-145 prostate cancer and MDA-MB-231 breast cancer cell lines were 2.97 and 3.26 μM, respectively.

Jeon and colleagues [146] developed monomeric STING agonists with amido benzimidazole and pyridine cores. Derivative **92** (Figure 16) demonstrated activity on human leukemia cell line THP-1VT with an IC_50_ of 3.1 μM. The pharmacokinetics of the lead compound **92** were investigated both in vivo and in vitro. Compound **92** exhibited 18.06% and 27.85% stability during the microsomal phase of the mouse and human liver, respectively. Plasma stability was found to be 99.9% in both cases. In addition, the plasma clearance was measured to be 5.73 L/h/kg and the half-life (T1/2) was 13.31 h. Hybrid **92** reduced tumor growth by 50.5% over 12 days when administered orally to syngeneic mice bearing breast cancer.

Ko and his team [147] obtained compound **93** (Figure 16), which effectively inhibited *FLT3* wild-type and *FLT3* D835Y mutated genes, showing IC_50_ values of 0.941 and 0.199 nM, respectively, in acute myeloid leukemia cells. Compound **93** has a central benzimidazole nucleus with an indazole ring on one side and a furan moiety on the other. In addition, Western blot assays revealed that derivative **93** inhibited STAT5 phosphorylation at a concentration of 1 nM upon treatment of MV4-11 myelomonocytic leukemia cells for 24 h, with a sub-nanomolar GI_50_ of 0.26 nM. Hybrid **93** was ten times more potent than the positive control gilteritinib (GI_50_: 2.90 nM).

Bradley et al. [148] designed a new selective *N*-terminal bromodomain BET using basic piperidine and benzimidazole as chemical probes. Compound **94** (Figure 16) exhibited a remarkable 1000-fold selectivity for the BD1 domain compared to BRD4 BD2, with an IC_50_ for hWB MCP-1 of 126 nM (pIC_50_: 6.9 nM) and an excellent water solubility of ≥0.52 µM.

Cui et al. [149] investigated new potent SYK inhibitors and their biological effects on hematological cancer cells. Compound **95** (Figure 16) exhibited robust inhibition of SYK and reduced PLCγ2 phosphorylation in MV4-11 myelomonocytic leukemia and RAMOS B-cell lymphoma cells at micromolar concentrations of 1.5 μM and 18.0 μM, respectively. Hybrid compound **95** had a terminal half-life of 3.79 h when administered orally and 6.49 h intravenously.

**Figure 17:**
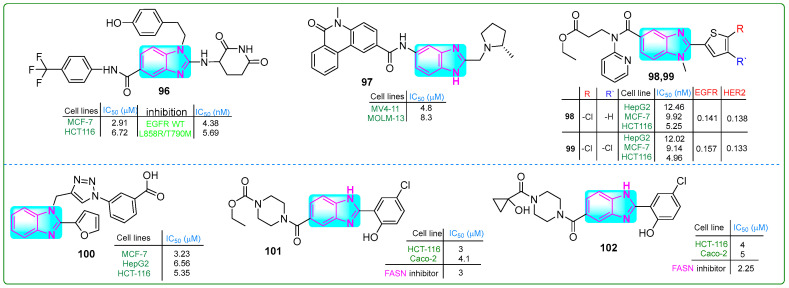
The structures of benzimidazole compounds **96**–**102**, which contain potential antitumor properties.

Theodore et al. [150] designed novel benzimidazole-based EFGR inhibitors and evaluated their efficacy against MCF-7 breast cancer and HCT-116 colon cancer cells. Compound **96** (Figure 17) exhibited the most potent inhibitory effects on EFGR/WT and EFGR/LR/TM, with IC_50_ values of 4.38 and 5.69 nM, respectively. It also suppressed the proliferation of MCF-7 and HCT-116 cancer cells at concentrations of 2.07 and 6.72 µM, respectively. Furthermore, its cytotoxicity on normal Vero cells was lower (IC_50_: 56.19 nM) than that of erlotinib (IC_50_: 18.69 nM).

Guo and colleagues [151] reported the synthesis of (S)-5-methyl-*N*-(2-((2-methylpyrrolidin-1-yl)methyl)-1*H*-benzo[d]imidazol-5-yl)-6-oxo-5,6-dihydrophenanthridine-2-carboxamide (**97**, (Figure 17)), which exhibited an IC_50_ of 15.4 nM for ENL inhibition. Subsequently, treatment of MOLM-13 and MV4-11 leukemia cells for 72 h with compound **97** inhibited the proliferation at concentrations of 8.3 μM and 4.8 μM, respectively. These results suggest that compound **97** was 7- and 9-fold more potent against these cancer cell lines than the positive control SGC-iMLLT.

Shinde and coworkers [152] designed new thiophene–pyridine–benzimidazole hybrids and evaluated their anticancer properties. Compounds **98** and **99** (Figure 17) exhibited the lowest IC_50_ values against the HepG2, MCF-7, and HCT116 cancer cell lines. Compound **99** showed IC_50_ values of 12.02 μM, 9.14 μM, and 4.96 μM, respectively, while compound **98** showed IC_50_ values of 12.46 μM, 9.92 μM, and 5.25 μM against the same cancer cells.

Alzahrani et al. evaluated a triazole–benzimidazole–oxazole hybrid [153]. Compound **100** (Figure 17) outperformed tamoxifen, chosen as a positive control, but was less effective than doxorubicin. MTT assay results indicated that compound **100** inhibited the growth of HepG-2, HCT-116, and MCF-7 cell lines at concentrations of 6.56, 5.30, and 3.23 μM, respectively. According to the docking assay, derivative **100** exhibited the highest free binding energy of −7.92 kcal/mol.

Singh and coworkers [154] developed new benzimidazole derivatives that inhibit the enzyme FASN, which plays a crucial role in palmitate biosynthesis, a process essential for cancer cell development and division. Compounds **101** and **102** (Figure 17) effectively blocked FASN, with IC_50_ values of 3 μM and 2.5 μM, respectively. Compound **101** exhibited the highest cytotoxic activity against HCT-116 and Caco-2 colon cancer cells, as well as MCF-7 breast cancer cells, at low doses of 3, 4.1, and 3.5 μM, while showing lower cytotoxicity (IC_50_ = 30.1 μM) against HEK 293 human embryonic kidney cells. In addition, these hybrids induced apoptosis in HCT-116 cells by decreasing Bcl-xl, increasing caspase-3 activity, and accumulating cells in the sub-G0/G1 cell cycle phase.

**Figure 18:**
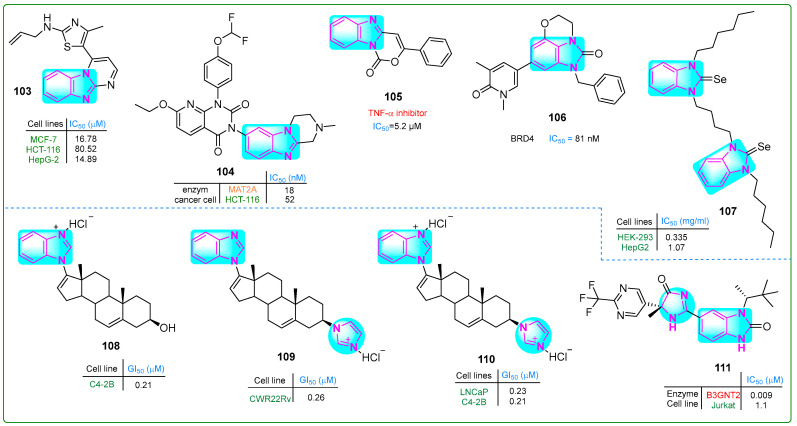
The structures of condensed and complex benzimidazole derivatives **103**–**111**, with antitumor potential.

Bondock et al. [155] prepared enaminones conjugated with benzimidazole and pyrimidine and evaluated their antitumor properties. Hybrid **103** (Figure 18) effectively inhibited MCF-7 and HepG-2 carcinoma cells at concentrations of 16.78 and 14.89 μM, respectively. Furthermore, compound **103** did not show cytotoxic effects when normal REP1 retinal pigment epithelial cells were treated for 48 h.

Zhang et al. [156] synthesized a novel drug candidate that inhibited methionine adenosyltransferase 2A, thereby suppressing cancer cell growth. Compound **104** (Figure 18) blocked MAT2A with an IC_50_ value of 26 nM and induced 52% cell death in HCT-116 cells after incubation at a dose of 50 mg/kg for 28 days. Pharmacokinetic analysis of derivative **104** revealed that the maximum plasma concentration was 0.67 μg/L with a half-life of 2.98 h.

Rao and colleagues [157] synthesized novel tricyclic benzimidazoxazinone compounds as TNF-α inhibitors. Some of these compounds showed significantly lower IC_50_ values than the positive control, thalidomide (IC_50_ ~ 197.3 µM). The compound with the lowest IC_50_, derivative **105** (5.2 μM) (Figure 18), was suggested for further pharmacological evaluation. The mouse macrophage line RAW 264.7 was treated with hybrid **105** at concentrations of 100, 60, and 30 μM, leading to cell viability percentages of 75.13%, 79.52%, and 81.47%, respectively, along with a bioavailability score of 0.55.

Horai et al. [158] developed a new tricyclic benzimidazole derivative combined with pyran that showed antitumor activity. Compound **106** (Figure 18) inhibited BRD4, leading to the arrest of melanoma cell growth in vitro (IC_50_: 15 nM). When administered orally at a dose of 60 mg/kg twice daily for 14 days to an A375.S2 melanoma xenograft mouse model, tricyclic imidazole hybrid **106** reduced skin cancer cell proliferation by nearly 41% without a significant effect on body weight.

Hassan and others [159] synthesized dinuclear azolium selenium adducts and evaluated their biological activities. The anticancer activity of these scaffolds was evaluated against HepG2 hepatocellular carcinoma cells, human embryonic kidney cells (HEK-293), and normal human endothelial cells (EA.hy926) using the MTT assay. Compound **107** (Figure 18) exhibited the highest cytotoxicity, with IC_50_ values of 0.54 µM against HEK-293 cells and 1.73 µM against HepG2 cells. Additionally, compound **107** exhibited the lowest toxicity against the standard cell line EA.hy926 (IC_50_: 34.16 µM).

Thankan et al. [160] synthesized mono-HCl Gal salt (**108**) along with mono- and di-HCl VNPPP433-3β salts (**109** and **110**) (Figure 18) to inhibit degradative adhesion signaling, specifically AR/AR-V7 and Mnk1/2-eIF4E. Compounds **108**–**110** were orally bioavailable, safe, and remarkably effective against aggressive CWR22Rv1 prostate carcinoma cells and tumor xenografts. These compounds outperformed the positive controls, abiraterone and enzalutamide. Compound **108** exhibited IC_50_ values of 0.36, 0.21, and 0.56 nM against the LNCaP, C4-2B, and CWR22Rv prostate cancer cell lines, respectively. Hybrid **109** showed IC_50_ values of 0.28, 0.33, and 0.26 nM against the same cell types, while compound **110** exhibited IC_50_ values of 0.23, 0.21, and 0.28 nM.

Jackson et al. [161] described the preparation of new benzimidazole derivatives with chiral centers. Enzymatic studies indicated that compound **111** (Figure 18) had potential as an inhibitor of B3GNT2. Structural and in vitro analyses revealed IC_50_ values of 0.009 nM for derivative **111** against the B3GNT2 enzyme and 1.1 nM against Jurkat acute T-cell leukemia cells.

**Figure 19:**
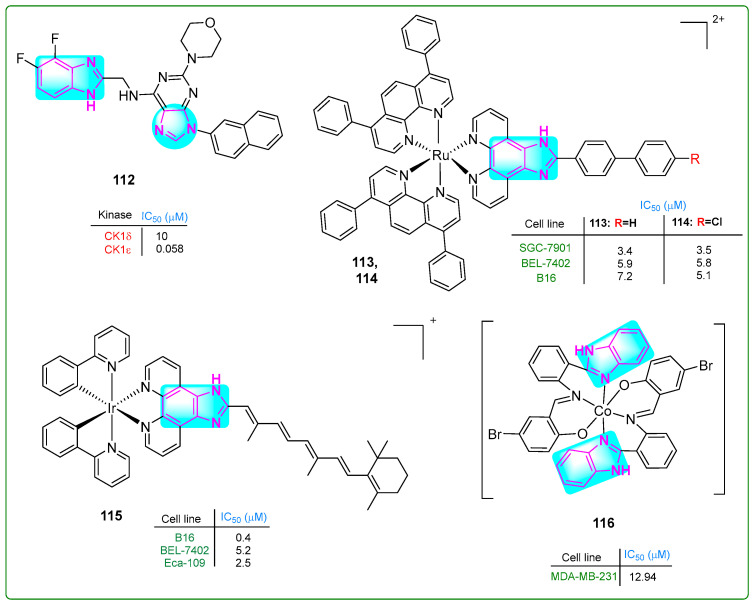
The benzimidazole complex derivatives **112**–**116** with antitumor activity.

Choi and colleagues [162] prepared new derivatives of benzimidazole–purine–morphine hybrids. Compound **112** (Figure 19) decreased the proliferation of bladder cancer cells by phosphorylating the catalytic domain of 4E-BP1, a target of casein kinase 1 (CK1e), at a concentration of 0.058 nM. The antiproliferative efficacy of **112** was measured in human bladder cancer cell lines (T24, 5637, UM-UC-3, and U2-OS) at concentrations of 256, 314, 540, and 373 nM, respectively.

Chen et al. [163] synthesized ruthenium compounds with a benzimidazole core as alternatives to the anticancer drug cisplatin. The compounds **113** and **114** (Figure 19) exhibited more significant cytotoxicity than cisplatin in human SGC-7901 gastric cancer, human BEL-7402 cervical cancer, and murine B16 melanoma cell lines, with IC_50_ values of 3.4 μM, 5.8 μM, and 7.2 μM for **113** and 3.5 μM, 5.8 μM, and 5.1 μM for **114**, respectively. Cisplatin had IC_50_ values of 5.7 μM, 15.2 μM, and 19.6 μM. Compounds **113** and **114** induced cell death through multiple mechanisms. These included autophagy, ferroptosis, apoptosis, mitochondrial dysfunction, and activation of caspase 3.

A new iridium (Ir) complex with a benzimidazole ring was developed by Yuan et al. [164]. The Ir complex **115** (Figure 19) effectively inhibited tumor growth in vivo at a dose of 5 mg/kg, resulting in a 71.67% inhibition rate. Notably, the Ir complex **115** displayed considerable cytotoxic effects against B16 melanoma and Eca-109 esophageal squamous cell carcinoma cells, with low IC_50_ values of 0.4 μM, 5.2 μM, and 2.5 μM, respectively.

Hou and colleagues [165] identified new Schiff-based benzimidazole–purine–morphine hybrid compounds with two benzimidazole rings. The in vitro MTT assay revealed that the cobalt(III) complex **116** (IC_50_: 12.94 μM) (Figure 19) exhibited more potent antiproliferative activity than cisplatin (IC_50_: 16.8 μM) in MDA-MB-231 breast cancer cells. The complex **116** exhibited comparable activity to cisplatin against A549 lung adenocarcinoma cells, with an IC_50_ of 13.19 μM. It exhibited reduced activity against the HeLa-derived cell lines CNE-2Z and SMMC-7721, with IC_50_ values of 28.23 and 34.46 μM, respectively. Benzimidazole complex **116** increased the intracellular free oxygen level, arrested the cell cycle in the G0/G1 phase, and induced apoptosis.

## 10. Structure–Activity Relationships (SARs)

In total, this review highlights 116 imidazole-containing hybrids. The most influential fragments, enhanced groups, and pharmacophore substituents are presented in Table 1. The key substituents affecting the anticancer properties of the imidazole hybrids include the methoxy (-OCH_3_) group and chlorine and fluorine halogens. When the methylene bridge and amide group were optimized for potency, piperazine or morpholine fragments affected the solubility of most hybrid molecules. Furthermore, the activity was influenced by heterocyclic rings such as quinoline, quinazoline, pyrazole, indolinone, and metals, especially in complex hybrids.

## 11. Conclusions and Perspectives

In summary, lead compounds featuring the imidazole motif show promise as effective drug candidates in the future, whether in their existing form or after necessary chemical and biological modifications. This review demonstrates that most synthesized imidazole hybrids exhibit encouraging anticancer activity. A key point is that positioning the imidazole core within the hybrid molecule significantly affects potency and selectivity. When the imidazole core was situated in the side chain, the hybrid molecule demonstrated efficacy in specific cancer cell types, particularly breast cancer cells. When it was located in the center or condensed with other heterocycles, it inhibited cell lines of other tumor types, particularly colon cancer cells. This highlights an essential aspect of this scaffold’s nature. These compounds enabled chemical interactions between imidazole and various enzymes. They effectively oriented the corresponding side rings, such as the fused ring in the imidazole hybrid system, toward the binding sites of proteins, enzymes, and amino acids. The unique properties of imidazole in these hybrid systems offer considerable advantages in drug design and delivery. While earlier bioavailability, solubility, and metabolic properties of imidazole-tethered hybrids posed significant challenges, recent advances address these limitations and reaffirm these scaffolds as a privileged class of heterocycles. Although there have been significant advancements in the physicochemical properties of common imidazole hybrids, including selectivity and water solubility, results from in vivo assays indicate that further development is necessary, particularly for imidazole metal complexes. Functionalizing these compounds into metal complexes through further chemical modification enhances solubility and could improve uptake by cancer cells. Imidazole-containing lead compounds, as essential family members of azoles, may serve as privileged synthons in pharmaceutical sciences and medicinal chemistry.

## Figures and Tables

**Table 1:** SARs of imidazole hybrids **1**–**116**.

Lead Compounds	Rings and Fragments Influencing Activity	Groups/Sections Enhancing Activity	Pharmacophore Substituents	Lead Compounds	Rings and Fragments Influencing Activity	Groups/Sections Enhancing Activity	Pharmacophore Substituents
**1**	1,2,4-Oxadiazole	Azomethine	-OCH_3_	**59**	Naphtho/imidazole	Phenyl	-NO_2_
**2**	Pyrazole	-NH-CH_2_-	-OCH_3_	**60**	Naphtho/imidazole	Phenyl	-OCH_3_
**3**	Pyrazole	-NH-CH_2_-	-F	**61**	Naphtho/imidazole	Phenyl	-Cl
**4**	Piperazine-2,5-dione	Indole	-Bn	**62**	Anthra/imidazolium	Carbonyl	-CH_3_
**5**	Piperazine	Sulfonamide	-CF_3_	**63**	Co	Amide	-CH_3_
**6**	Benzofuran, triazole	Amide	-OCH_3_	**64**	Fe	Phenylphosphane	-Bn
**7**	Indolin-2-one	-NH-CH_2_-	-OCH_3_	**65**	Ag	Imine	-Br
**8**	Quinazoline, indolinone	Amide	-Cl, -F	**66**	Au	Imine	-Cl
**9**	Pyrimidine, thiophene	Amide	-F, -Cl, -Bn	**67**	Pt	Imine	-CH_3_
**10**	Pyrazine, triazole	Amide	-F, -Bn	**68**	Pt	Imine	-Cl, -OCH_3_
**11**	Azomethine, thione	Imine	-CH_3_	**69**	Ru	Azomethine	-OH
**12**	Thione	Carbonyl	-Cl, -Bn	**70**	Au	poly-phenyl	-C_2_H_5_
**13**	Ethoxy/ethoxy	Amide	-F	**71**	Pt, pyrimidine, purine	Carbonyl	-CH_3_
**14**	Ethoxy/ethoxy	Amide	-Cl	**72**	Au	Methylene bridge	-OCH_3_
**15**	Triazole	Phenoxy	-Cl, -OH	**73**	Chromenone	Oxadiazole	-CH_3_
**16**	Piperazine	Amide	-CF_3_	**74**	Triazine, piperazine	Imine, morpholine	-F
**17**	Quinoline	Oxazepinone	-CF_3_, -OCH_3_	**75**	Pyrrole, thiophene	Amide	-CH_3_, -OCH_3_
**18**	Cyclopenta/phenanthren	Amide	-F	**76**	Piperidine	Sulfonamide	-CF_3_
**19**	Pyrimidine	Amide	-CF_3_	**77**	Thiazole, pyridine	Amide	-CH_3_
**20**	Triazole, piperazine	Amide	-Cl	**78**	Thienopyrimidine	Morpholine	-S(O)_2_-CH_3_
**21**	Quinoline	3,4,5-tri-OCH_3_	-OCH_3_	**79**	Thiazole	Thioimidazole	-CH_2_-
**22**	di-Phenyl	Ph-4-OCH_3_	-OCH_3_	**80**	Quinazoline	Amide	Bn
**23**	Furyl	Ph-4-CH_3_	-CH_3_	**81**	Chromenone	Thioimidazole	-OCH_3_
**24**	Pyridine	Carbonyl	-OCH_3_	**82**	Triazine, piperazine	Morpholine, Amide	-Cl, -F, OCH_3_
**25**	Piperazine	Biphenyl	-CF_3_	**83**	Pyrimidine, Pyrazole	Amide	-Cl, -CH_3_
**26**	Pyrazole, pyridine	-NH-CH_2_-	-F	**84**	*Bis*-benzyl	Amide	-OH
**27**	Thieno[3,4-*d*]imidazole	Sulfamide	-[-CH_2_]-	**85**	Pyrimidine	Piperidine	-I
**28**	Imidazo[2,1-*b*]thiazole	*tert*-Bu-NH_2_	-OCH_3_	**86**	Phenylimidazole	Ester	-Cl
**29**	Imidazo/thiadiazole	*tert*-Bu	-F	**87**	Phenylimidazole	Ester	-Bu
**30**	di-Benzyl	Amide	-OCH_3_	**88**	Oxazole, pyrazole	Methylene bridge, amide	-CF_3_
**31**	Imidazo/triazole	Chromen	-F	**89**	Pyrazole	Methylene bridge, amide	-F, -OCH_3_
**32**	Imidazo/triazole	Chromen	-OCH_3_	**90**	Quinoline	Amide	-NH_2_
**33**	Imidazo/pyridine, Pyrimidine	Amide	-OCH_3_	**91**	Thiadiazole,	Piperazine	-F, -CF_3_
**34**	Imidazo/pyridine	Methylene bridge	-COOH	**92**	Nicotinamide	Methylene bridge, Amide	-CH_3_
**35**	Imidazo/pyridine	Methylene bridge	-COOH, -OH	**93**	Indazole, furan	Amide	-NH_2_
**36**	Imidazo/pyridazine	Amide	-F	**94**	Piperidine	Pyridinone	-CH_3_
**37**	Imidazo/pyridazine	Oxazole	-N-(Et)_2_	**95**	Pyrrolopyrimidine, piperazine	Pyrazole	-Pr
**38**	Imidazo/pyridazine; triazole	Morpholine, sulfamide	-[-CH_2_]-, -F	**96**	Dioxopiperidine	Amide	-CF_3_
**39**	Imidazo/pyridine, indoline	Methylene bridge	-S(O)_2_-CH_3_	**97**	Pyrrolidine	Amide	-CH_2_-, -CH_3_
**40**	Imidazo/pyridine, quinoline	Methylene bridge	-S(O)_2_-CH_3_	**98**	Thiophene	Methylene bridge, amide	-Cl
**41**	Imidazo/pyridine, indole	3,4,5-tri-OCH_3_	-OCH_3_	**99**	Thiophene	Amide	-Cl
**42**	Imidazo/pyridazine, piperazine	Amide, pyrrolidine	-CF_3_	**100**	Triazole	Methylene bridge	-COOH
**43**	Imidazo/pyridazine, piperazine	Methylene bridge	-CH_3_	**101**	Piperazine	Carbonyl	-Cl
**44**	Purine, piperazine	Sulfamide	-CH_3_	**102**	Piperazine	Carbonyl	-Cl
**45**	Purine	Methylene bridge, phosphone	-Cl	**103**	Thiazole	Imine	-CH_3_
**46**	Purine, thiophene	Methylene bridge	-OH	**104**	Pyridopyrimidine	Methylpiperazine	-CH_3_
**47**	Purine	Oxadiazole	-Cl, -CF_3_	**105**	Chromen	Carbonyl	-Ph
**48**	Purine, piperazine	Sulfone	-OCH_3_	**106**	Oxo-dihydropyrimidine	Morpholine, carbonyl	-Bn
**49**	Imidazo/tetrazinone	Thiazole	-Propynyl	**107**	*Bis*-benzimidazole	Methylene bridge	-CH_3_
**50**	Benzamide, pyrrole	Sulfamide	-Cl	**108**	Galeterone (benzimidazole portion)	Galeterone (steroid portion)	-CH_3_, -OH
**51**	Pyrrole	Amide, Methylene bridge	-CH_3_	**109**	Galeterone (benzimidazole portion)	Galeterone (steroid portion)	-CH_3_
**52**	Purine, Imidazo/thiazole	Methylene bridge	-CH_3_	**110**	Galeterone (benzimidazole portion)	Galeterone (steroid portion)	-CH_3_
**53**	Imidazo/oxazole	3,4,5-tri-OCH_3_	-Br, -OCH_3_	**111**	Pyrimidine	Carbonyl	-CF_3_
**54**	Purine	Phosphone	-F	**112**	*Bis*-benzimidazole	Morpholine	-F
**55**	Purine	Thione	-CH_3_, -OH	**113**	Ru	Biphenyl	-Cl
**56**	Purine	Thiadiazine	-Ph	**114**	Ru	Biphenyl	-Ph
**57**	Quinazoline	Amine	-OCH_3_	**115**	Ir	-[-CH = CH-]-	-CH_3_
**58**	Purine	Thiazepine	-Cl	**116**	Co	Azomethine	-Br

## References

[1] Giaquinto A.N., Miller K.D., Tossas K.Y., Winn R.A., Jemal A., Siegel R.L. (2022). Cancer statistics for African American/Black People 2022. CA Cancer J. Clin..

[2] Parshuram Satpute D., Shirwadkar U., Kumar Tharalla A., Dattatray Shinde S., Nikhil Vaidya G., Joshi S., Patel Vatsa P., Jain A., Singh A.A., Garg R. (2023). Discovery of fluorinated 2-Styryl 4(3H)-quinazolinone as potential therapeutic hit for oral cancer. Bioorg. Med. Chem..

[3] Das B.C., Lepe J.J., Adil Shareef M., Lomeli N., Das S., Bota D.A. (2023). Identification of new hit to lead magmas inhibitors as potential therapeutics for glioblastoma. Bioorg. Med. Chem. Lett..

[4] Lin K., Zhang X., Dai X., Ma L., Bozorov K., Guo H., Huang G., Cao J. (2021). Synthesis and Anticancer Activity of Podophyllotoxin Derivatives. Chem. Nat. Compd..

[5] Agboyibor C., Dong J., Effah C.Y., Drokow E.K., Ampomah-Wireko M., Pervaiz W., Sangmor A., Ma X., Li J., Liu H.-M. (2023). Epigenetic compounds targeting pharmacological target lysine specific demethylase 1 and its impact on immunotherapy, chemotherapy and radiotherapy for treatment of tumor recurrence and resistance. Biomed. Pharmacother..

[6] Zhu R.Y., Bozorov K., Aisa H.A., Zhao J.Y. (2025). Design and Synthesis of Rupestonic Acid Derivatives and Assessment of Their Cytotoxic Activity. Chem. Nat. Compd..

[7] Lang K.D., Kaur R., Arora R., Saini B., Arora S. (2020). Nitrogen-Containing Heterocycles as Anticancer Agents: An Overview. Anticancer Agents Med. Chem..

[8] Martins P., Jesus J., Santos S., Raposo L.R., Roma-Rodrigues C., Baptista P.V., Fernandes A.R. (2015). Heterocyclic Anticancer Compounds: Recent Advances and the Paradigm Shift towards the Use of Nanomedicine’s Tool Box. Molecules.

[9] Lu T., Nie L., Tang D., Bozorov K., Zhao J., Aisa H.A. (2024). Synthesis of tricyclic pyrazolopyrimidine arylidene ester derivatives and their cytotoxic and molecular docking evaluations. J. Heterocycl. Chem..

[10] Ruzi Z., Bozorov K., Nie L., Zhao J., Akber Aisa H. (2023). Discovery of novel (E)-1-methyl-9-(3-methylbenzylidene)-6,7,8,9-tetrahydropyrazolo [3,4-d]pyrido[1,2-a]pyrimidin-4(1H)-one as DDR2 kinase inhibitor: Synthesis, molecular docking, and anticancer properties. Bioorg. Chem..

[11] Ruzi Z., Bozorov K., Nie L., Zhao J., Aisa H.A. (2022). Novel pyrazolo[3,4-d]pyrimidines as potential anticancer agents: Synthesis, VEGFR-2 inhibition, and mechanisms of action. Biomed. Pharmacother..

[12] Zeng Y., Nie L., Bozorov K., Ruzi Z., Song B., Zhao J., Aisa H.A. (2022). 2-substituted tricyclic oxazolo[5,4-d]pyrimidine library: Design, synthesis, and cytotoxicity activity. J. Heterocycl. Chem..

[13] Song B., Nie L., Bozorov K., Kuryazov R., Zhao J., Aisa H.A. (2023). Design, combinatorial synthesis and cytotoxic activity of 2-substituted furo[2,3-d]pyrimidinone and pyrrolo[2,3-d]pyrimidinone library. Mol. Divers..

[14] Song B., Nie L., Bozorov K., Kuryazov R., Aisa H.A., Zhao J. (2023). Parallel synthesis of condensed pyrimidine-thiones and their antitumor activities. Res. Chem. Intermed..

[15] Song B., Nie L., Bozorov K., Niu C., Kuryazov R., Akber Aisa H., Zhao J. (2023). Furo[2,3-d]pyrimidines as Mackinazolinone/Isaindigotone Analogs: Synthesis, Modification, Antitumor Activity, and Molecular Docking Study. Chem. Biodivers..

[16] Zeng Y., Nie L., Niu C., Mamatjan A., Bozorov K., Zhao J., Aisa H.A. (2022). Synthesis and Biological Activities of Dihydrooxazolo[5,4-d]-pyrrolo[1,2-a]pyrimidinones. Chin. J. Org. Chem..

[17] Nasrullaev A., Bozorov K., Bobakulov K., Zhao J., Nie L.F., Turgunov K.K., Elmuradov B., Aisa H.A. (2019). Synthesis, characterization, and antimicrobial activity of novel hydrazone-bearing tricyclic quinazolines. Res. Chem. Intermed..

[18] Nie L.F., Bozorov K., Huang G., Zhao J., Niu C., Aisa H.A. (2018). Design, synthesis, and toward a side-ring optimization of tricyclic thieno[2,3-d]pyrimidin-4(3H)-ones and their effect on melanin synthesis in murine B16 cells. Phosphorus Sulfur Silicon Relat. Elem..

[19] Nie L.F., Huang G., Bozorov K., Zhao J., Niu C., Sagdullaev S.S., Aisa H.A. (2018). Diversity-oriented synthesis of amide derivatives of tricyclic thieno[2,3-d]pyrimidin-4(3H)-ones and evaluation of their influence on melanin synthesis in murine B16 cells. Heterocycl. Commun..

[20] Bozorov K.A., Mamadalieva N.Z., Elmuradov B.Z., Triggiani D., Egamberdieva D., Tiezzi A., Aisa H.A., Shakhidoyatov K.M. (2013). Synthesis of substituted thieno[2, 3-D]pyrimidin-4-ones and their testing for evaluation of cytotoxic activity on mammalian cell models. J. Chem..

[21] Liu F., Hou X., Nie L.F., Bozorov K., Decker M., Huang G. (2018). A Convenient One-pot Synthesis of 2,3-Disubstituted Thieno[2, 3-d]pyrimidin-4(3 H)-ones from 2 H -Thieno[2, 3-d ][1,3]oxazine-2,4(1 H)-diones, Aromatic Aldehydes and Amines. SynOpen.

[22] Elmuradov B.Z., Bozorov K.A., Shakhidoyatov K.M. (2011). Thieno[2,3-d]pyrimidin-4-ones 1. condensation of 2,3-dimethyl- and 2,3-tri-, 2,3-tetra-, and 2,3-pentamethylene-7,8-dihydro-pyrrolo[1,2-a]thieno[2, 3-d]pyriminidin-4(6H)-ones with aromatic aldehydes and furfural. Chem. Heterocycl. Compd..

[23] Bozorov K., Zhao J.Y., Aisa H.A. (2016). Recent advances in ipso-nitration reactions. ARKIVOC.

[24] Nie L.F., Bozorov K., Niu C., Huang G., Aisa H.A. (2017). Synthesis and biological evaluation of novel sulfonamide derivatives of tricyclic thieno[2,3-d]pyrimidin-4(3H)-ones on melanin synthesis in murine B16 cells. Res. Chem. Intermed..

[25] Zeng Y., Nie L., Liu L., Niu C., Li Y., Bozorov K., Zhao J., Shen J., Aisa H.A. (2022). Design, synthesis, in vitro evaluation of a new pyrrolo[1,2-a]thiazolo[5,4-d]pyrimidinone derivatives as cholinesterase inhibitors against Alzheimer’s disease. J. Heterocycl. Chem..

[26] Bozorov K., Elmuradov B., Shakhidoyatov K., Aisa H.A., Tashkhodjaev B. (2013). 2,3-Trimethylene-7,8-dihydropyrrolo[1,2-a]thieno[2,3-d]pyrimidin-4(6H)-one. Acta Crystallogr. Sect. E Struct. Rep. Online.

[27] Elmuradov B.Z., Bozorov K.A., Okmanov R.Y., Tashkhodjaev B., Shakhidoyatov K.M. (2011). 2-Methyl-4-oxo-6,7,8,9-tetrahydro-thieno[2′,3′:4,5] pyrimidino-[1,2-a]pyridine-3-carboxylic acid. Acta Crystallogr. Sect. E Struct. Rep. Online.

[28] Boiani M., González M. (2005). Imidazole and benzimidazole derivatives as chemotherapeutics agents. Mini-Rev. Med. Chem..

[29] Ali I., Lone M.N., Aboul-Enein H.Y. (2017). Imidazoles as potential anticancer agents. MedChemComm.

[30] Wang X., Wang Y., Liu X., He T., Li L., Wu H., Zhou S., Li D., Liao S., Xu P. (2021). Imidazole hydrochloride promoted synthesis of 3,5-disubstituted-1,2,4-oxadiazoles. Tetrahedron.

[31] Jin Z. (2003). Muscarine, imidazole, oxazole, and thiazole alkaloids. Nat. Prod. Rep..

[32] Babaev E.V., Atta ur R. (2017). Chapter 2-2-Aminoimidazoles: Synthesis by Ring Transformation Reactions. Studies in Natural Products Chemistry.

[33] Ruzi Z., Nie L., Bozorov K., Zhao J., Aisa H.A. (2021). Synthesis and anticancer activity of ethyl 5-amino-1-N-substituted-imidazole-4-carboxylate building blocks. Arch. Pharm..

[34] Guo H., Nie L., Bozorov K., Aisa H.A., Zhao J. (2022). Synthesis and Antitumor Activity of Novel Linear Tricyclic Compounds Derived from Purine. Heterocycles.

[35] Peytam F., Emamgholipour Z., Mousavi A., Moradi M., Foroumadi R., Firoozpour L., Divsalar F., Safavi M., Foroumadi A. (2023). Imidazopyridine-based kinase inhibitors as potential anticancer agents: A review. Bioorg. Chem..

[36] Andreani A., Burnelli S., Granaiola M., Leoni A., Locatelli A., Morigi R., Rambaldi M., Varoli L., Calonghi N., Cappadone C. (2008). New Antitumor Imidazo[2,1-b]thiazole Guanylhydrazones and Analogues^1^. J. Med. Chem..

[37] Andreani A., Burnelli S., Granaiola M., Leoni A., Locatelli A., Morigi R., Rambaldi M., Varoli L., Farruggia G., Stefanelli C. (2006). Synthesis and Antitumor Activity of Guanylhydrazones from 6-(2,4-Dichloro-5-nitrophenyl)imidazo[2,1-b]thiazoles and 6-Pyridylimidazo[2,1-b]thiazoles. J. Med. Chem..

[38] Andreani A., Granaiola M., Leoni A., Locatelli A., Morigi R., Rambaldi M., Lenaz G., Fato R., Bergamini C., Farruggia G. (2005). Potential Antitumor Agents. 37. Synthesis and Antitumor Activity of Guanylhydrazones from Imidazo[2,1-b]thiazoles and from the New Heterocyclic System Thiazolo[2‘,3‘:2,3]imidazo[4,5-c]quinoline. J. Med. Chem..

[39] Andreani A., Burnelli S., Granaiola M., Leoni A., Locatelli A., Morigi R., Rambaldi M., Varoli L., Calonghi N., Cappadone C. (2008). Antitumor Activity of New Substituted 3-(5-Imidazo[2,1-b]thiazolylmethylene)-2-indolinones and 3-(5-Imidazo[2,1-b]thiadiazolylmethylene)-2-indolinones: Selectivity against Colon Tumor Cells and Effect on Cell Cycle-Related Events. J. Med. Chem..

[40] Del Vecchio A., Rosadoni E., Ballerini L., Cuzzola A., Lipparini F., Ronchi P., Guariento S., Biagetti M., Lessi M., Bellina F. (2024). Transition Metal-Driven Selectivity in Direct C−H Arylation of Imidazo[2,1-b]Thiazole. ChemistryOpen.

[41] Tominaga T., Adachi I., Sasaki Y., Tabei T., Ikeda T., Takatsuka Y., Toi M., Suwa T., Ohashi Y. (2003). Double-blind randomised trial comparing the non-steroidal aromatase inhibitors letrozole and fadrozole in postmenopausal women with advanced breast cancer. Ann. Oncol..

[42] Patel N., Neupane R., Balaji S., Tiwari A.K., Ray S.D., Wexler P. (2024). Dacarbazine. Encyclopedia of Toxicology.

[43] Erba H.P., Montesinos P., Kim H.-J., Patkowska E., Vrhovac R., Žák P., Wang P.-N., Mitov T., Hanyok J., Kamel Y.M. (2023). Quizartinib plus chemotherapy in newly diagnosed patients with FLT3-internal-tandem-duplication-positive acute myeloid leukaemia (QuANTUM-First): A randomised, double-blind, placebo-controlled, phase 3 trial. Lancet.

[44] Beck D.E., Agama K., Marchand C., Chergui A., Pommier Y., Cushman M. (2014). Synthesis and biological evaluation of new carbohydrate-substituted indenoisoquinoline topoisomerase I inhibitors and improved syntheses of the experimental anticancer agents indotecan (LMP400) and indimitecan (LMP776). J. Med. Chem..

[45] Shu L., Wang D., Nannapaneni S., Sun Y., Griffith C.C., Wang X., Chen Z., Patel M., El-Deiry M., Shin D.M. (2021). Tipifarnib enhances anti-EGFR activity of cetuximab in non-HRas mutated head and neck squamous cell carcinoma cancer (HNSCC). Oral Oncol..

[46] Menisy G.M., Zakaria S., Suddek G.M. (2022). Nilotinib alleviated acetaminophen-induced acute hepatic injury in mice through inhibiting HIF-1alpha/VEGF-signaling pathway. Int. Immunopharmacol..

[47] Gao Y., Ding Y., Tai X.-r., Zhang C., Wang D. (2023). Ponatinib: An update on its drug targets, therapeutic potential and safety. Biochim. Biophys. Acta.

[48] Shaveta, Mishra S., Singh P. (2016). Hybrid molecules: The privileged scaffolds for various pharmaceuticals. Eur. J. Med. Chem..

[49] Mancini I., Vigna J., Sighel D., Defant A. (2022). Hybrid Molecules Containing Naphthoquinone and Quinolinedione Scaffolds as Antineoplastic Agents. Molecules.

[50] Rosadoni E., Bombonato E., Del Vecchio A., Guariento S., Ronchi P., Bellina F. (2023). Direct Decarboxylative C-2 Alkylation of Azoles through Minisci-Type Coupling. J. Org. Chem..

[51] Duncton M.A.J. (2011). Minisci reactions: Versatile CH-functionalizations for medicinal chemists. MedChemComm.

[52] Proctor R.S.J., Phipps R.J. (2019). Recent Advances in Minisci-Type Reactions. Angew. Chem. Int. Ed..

[53] Minisci F., Bernardi R., Bertini F., Galli R., Perchinummo M. (1971). Nucleophilic character of alkyl radicals—VI: A new convenient selective alkylation of heteroaromatic bases. Tetrahedron.

[54] Zhang W., Nie L., Bozorov K., Aisa H.A., Zhao J. (2023). Synthesis of Diethyl 2,5-Diaminothiophene-3,4-dicarboxylate Derivatives and Antitumor Activity Study. Chin. J. Org. Chem..

[55] Kuryazov R.S., Mukhamedov N.S., Dushamov D.A., Okmanov R.Y., Shakhidoyatov K.M., Tashkhodzaev B. (2010). Quinazolines. 3*. synthesis of 6-bromo-8-chloro- sulfonylquinazoline- 2,4(1H,3H)-dione and its interaction with nucleophilic reagents. Chem. Heterocycl. Compd..

[56] Scopus Database. www.scopus.com.

[57] Tolomeu H.V., Fraga C.A.M. (2023). Imidazole: Synthesis, Functionalization and Physicochemical Properties of a Privileged Structure in Medicinal Chemistry. Molecules.

[58] Rani N., Singh R., Kumar P. (2023). Imidazole and Derivatives Drugs Synthesis: A Review. Curr. Org. Synth..

[59] Poyraz S., Yıldırım M., Ersatir M. (2024). Recent pharmacological insights about imidazole hybrids: A comprehensive review. Med. Chem. Res..

[60] Kumar A., Kaushal A., Verma P.K., Gupta M.K., Chandra G., Kumar U., Yadav A.K., Kumar D. (2024). An insight into recent developments in imidazole based heterocyclic compounds as anticancer agents: Synthesis, SARs, and mechanism of actions. Eur. J. Med. Chem..

[61] Ghara A., Matada G.S.P., Pramanick P., Sharma U.R., Metikurki B., Dhiwar P.S. (2025). Exploring the molecular insights of imidazole and benzimidazole scaffold for cancer therapy: Synthesis, in-vitro cytotoxicity and SAR studies. J. Mol. Struct..

[62] Lavunuri S., Nadh R.V., Rapeti S.K. (2024). Anti-Proliferative, Anti-EGFR and In Silico Studies of a Series of New Imidazole Tethered 1,2,4-Oxadiazoles. Polycycl. Aromat. Compd..

[63] Yevale D.B., Teraiya N., Lalwani T.D., Ameta R.K., Sangani C.B. (2023). A novel class of pyrazole analogues as aurora kinase A inhibitor: Design, synthesis, and anticancer evaluation. Bioorg. Chem..

[64] Fang S., Bi S., Li Y., Tian S., Xu H., Fu L., Wang S., Tang Y., Qiu P. (2023). Design, synthesis and anti-tumor evaluation of plinabulin derivatives as potential agents targeting β-tubulin. Bioorg. Med. Chem. Lett..

[65] Kardile R.A., Sarkate A.P., Lokwani D.K., Tiwari S.V., Azad R., Thopate S.R. (2023). Design, synthesis, and biological evaluation of novel quinoline derivatives as small molecule mutant EGFR inhibitors targeting resistance in NSCLC: In vitro screening and ADME predictions. Eur. J. Med. Chem..

[66] Gariganti N., Loke S.K., Pagadala E., Chinta P., Poola B., Chetti P., Bansal A., Ramachandran B., Srinivasadesikan V., Kottalanka R.K. (2023). Design, synthesis, anticancer activity of new amide derivatives derived from 1,2,3-triazole-benzofuran hybrids: An insights from molecular docking, molecular dynamics simulation and DFT studies. J. Mol. Struct..

[67] Qin Q., Fu Q., Wang X., Lv R., Lu S., Guo Z., Wu T., Sun Y., Sun Y., Liu N. (2023). Design, synthesis and biological evaluation of novel indolin-2-one derivatives as potent second-generation TRKs inhibitors. Eur. J. Med. Chem..

[68] Cheng W., Li S., Han S., Miao R., Wang S., Liu C., Wei H., Tian X., Zhang X. (2023). Design, synthesis and biological evaluation of the tumor hypoxia-activated PROTACs bearing caged CRBN E3 ligase ligands. Bioorg. Med. Chem..

[69] Tsuji T., Tsunematsu H., Imanishi M., Denda M., Tsuchiya K., Otaka A. (2023). Enhanced tumor specific drug release by hypoxia sensitive dual-prodrugs based on 2-nitroimidazole. Bioorg. Med. Chem. Lett..

[70] Wang P., Zhu W.-T., Wang Y., Song S.-S., Xi Y., Yang X.-Y., Shen Y.-Y., Su Y., Sun Y.-M., Gao Y.-L. (2023). Identification of [1,2,4]Triazolo[4,3-a]pyrazine PARP1 inhibitors with overcome acquired resistance activities. Eur. J. Med. Chem..

[71] Malakar K., Sohtun W.P., Srinivasan V., Saravanan D., Velusamy M. (2023). Molecular design and synthesis of dithiocarbazate-based potential biomaterials: Crystal structure, apoptotic activity and protein binding studies. J. Mol. Struct..

[72] Hassan A.Y., El Deeb M.A., El-Zoghbi M.S., El-Sebaey S.A., Mohamed N.M. (2023). Novel thioxoimidazolidinone derivatives as dual EGFR and CDK2 inhibitors: Design, synthesis, anticancer evaluation with in silico study. J. Mol. Struct..

[73] Zhang G., Li S., Wang F., Jones A.C., Goldberg A.F.G., Lin B., Virgil S., Stoltz B.M., Deshaies R.J., Chou T.-F. (2021). A covalent p97/VCP ATPase inhibitor can overcome resistance to CB-5083 and NMS-873 in colorectal cancer cells. Eur. J. Med. Chem..

[74] Wang K., Chen L., Dai X., Ye Z., Zhou C., Zhang C.-J., Feng Z. (2023). Synthesis and structure-activity relationships of N-(3-(1H-imidazol-2-yl) phenyl)-3-phenylpropionamide derivatives as a novel class of covalent inhibitors of p97/VCP ATPase. Eur. J. Med. Chem..

[75] Zarenezhad E., Behmard E., Sadeghian I., Sadeghian S., Ghanbariasad A., Ghasemian A., Behrouz S., Zarenezhad A., Rad M.N.S. (2023). Synthesis, cytotoxic evaluation, molecular docking studies and molecular dynamic simulation of some metronidazole analogues. J. Mol. Struct..

[76] Wang X., Xu Z., Feng J., Pan G., He X., Lv M., Chen H., Jiang W., Ji J., Yang M. (2023). Synthesis and biological evaluation of novel aromatic amide derivatives as potential BCR-ABL inhibitors. Bioorg. Med. Chem. Lett..

[77] Teuscher K.B., Mills J.J., Tian J., Han C., Meyers K.M., Sai J., South T.M., Crow M.M., Van Meveren M., Sensintaffar J.L. (2023). Structure-Based Discovery of Potent, Orally Bioavailable Benzoxazepinone-Based WD Repeat Domain 5 Inhibitors. J. Med. Chem..

[78] Adams P.D., Grosse-Kunstleve R.W., Hung L.W., Ioerger T.R., McCoy A.J., Moriarty N.W., Read R.J., Sacchettini J.C., Sauter N.K., Terwilliger T.C. (2002). PHENIX: Building New Software for Automated Crystallographic Structure Determination. Acta Crystallogr. Sect. D Biol. Crystallogr..

[79] Li Y., Liu Y., Zhang Y., Wu Y., Xing Z., Wang J., Fan G.-H. (2023). Discovery of a First-in-Class CD38 Inhibitor for the Treatment of Mitochondrial Myopathy. J. Med. Chem..

[80] Xie H., Xu W., Liang J., Liu Y., Zhuo C., Zou X., Luo W., Xiao J., Lin Y., Chen L. (2023). Design, synthesis and evaluation of EZH2-based PROTACs targeting PRC2 complex in lymphoma. Bioorg. Chem..

[81] Giri P., Batra P.J., Kumari A., Hura N., Adhikary R., Acharya A., Guchhait S.K., Panda D. (2023). Development of QTMP: A promising anticancer agent through NP-Privileged Motif-Driven structural modulation. Bioorg. Med. Chem..

[82] Muhammed M.T., Er M., Akkoc S. (2023). Molecular modeling and in vitro antiproliferative activity studies of some imidazole and isoxazole derivatives. J. Mol. Struct..

[83] Çetiner G., Acar Çevik U., Celik I., Bostancı H.E., Özkay Y., Kaplancıklı Z.A. (2023). New imidazole derivatives as aromatase inhibitor: Design, synthesis, biological activity, molecular docking, and computational ADME-Tox studies. J. Mol. Struct..

[84] Ibrahim S.A., Al-Mhyawi S.R., Atlam F.M. (2023). New imidazole-2-ones and their 2-thione analogues as anticancer agents and CAIX inhibitors: Synthesis, in silico ADME and molecular modeling studies. Bioorg. Chem..

[85] Wang X.-D., Wang J.-X., Yu B.-Y., Zhang S.-Q., Hu M.-H. (2023). Non-fused imidazole-biphenyl analogs repress triple-negative breast cancer growth by mainly stabilizing the c-MYC G-quadruplex via a multi-site binding mode. Bioorg. Med. Chem..

[86] Kang B.-N., Kang H.-J., Kim S., Lee J., Lee J., Jeong H.-J., Jeon S., Shin Y., Yoon C., Han C. (2023). Synthesis and biological evaluation of N-(3-fluorobenzyl)-4-(1-(methyl-d3)-1H-indazol-5-yl)-5-(6-methylpyridin-2-yl)-1H-imidazol-2-amine as a novel, potent ALK5 receptor inhibitor. Bioorg. Med. Chem. Lett..

[87] Begines P., Bonardi A., Nocentini A., Gratteri P., Giovannuzzi S., Ronca R., Tavani C., Luisa Massardi M., López Ó., Supuran C.T. (2023). Design and synthesis of sulfonamides incorporating a biotin moiety: Carbonic anhydrase inhibitory effects, antiproliferative activity and molecular modeling studies. Bioorg. Med. Chem..

[88] Singh R., Kumar R., Roy A., Behera P.M., Atri A.K., Kumar K., Manna D., Dixit A., Patil M.T., Mankamna Kumari R. (2023). Imidazo[2,1-b]thiazole based indoleamine-2,3-dioxygenase 1 (IDO1) inhibitor: Structure based design, synthesis, bio-evaluation and docking studies. Bioorg. Med. Chem. Lett..

[89] Khamees H.A., Srinivas M.S., Nagaraja O., Madegowda M., Vahini V., Chaluvaiah K., Dasappa J.P., Warad I. (2023). Studies on New Imidazo[2,1-b][1,3,4]thiadiazole Derivatives: Molecular Structure, Quantum Chemical Computational, and In silico Study of Inhibitory Activity Against Pim-1 Protein by using Molecular Modelling Methods and ADMET Profiling. J. Mol. Struct..

[90] Fu P.-P., Wang Q., Zhang Q., Jin Y., Liu J., Chen K.-X., Guo Y.-W., Liu S.-H., Li X.-W. (2023). Bioactivity-Driven Synthesis of the Marine Natural Product Naamidine J and Its Derivatives as Potential Tumor Immunological Agents by Inhibiting Programmed Death-Ligand 1. J. Med. Chem..

[91] Samala R., Nukala S.K., Manchal R., Nagavelli V.R., Narsimha S. (2023). Synthesis and biological evaluation of coumarine-imidazo[1,2-c][1,2,3]triazoles: PEG-400 mediated one-pot reaction under ultrasonic irradiation. J. Mol. Struct..

[92] Liu B., Gao F., Zhao H., Yuan S., Peng X., Zhang P., Wang J., Zhang T., Duan M., Guo Y. (2023). Discovery of YK-029A, a novel mutant EGFR inhibitor targeting both T790 M and exon 20 insertion mutations, as a treatment for NSCLC. Eur. J. Med. Chem..

[93] Imai Y., Suzuki R., Wakasugi D., Matsuda D., Tanaka-Yamamoto N., Ohki Y., Mima M., Endo M., Tabata R., Matsuzawa H. (2023). Structure-based lead optimization to improve potency and selectivity of a novel tetrahydroimidazo[1,2-a]pyridine-5-carboxylic acid series of heparanase-1 inhibitor. Bioorg. Med. Chem..

[94] Imai Y., Suzuki R., Matsuda D., Tanaka-Yamamoto N., Ohki Y., Tabata R., Kato S., Sugisaki M., Fujimoto N., Fukunaga T. (2024). Discovery of a novel tetrahydroimidazo[1,2-a]pyridine-5-carboxylic acid derivative as a potent and selective heparanase-1 inhibitor utilizing an improved synthetic approach. Bioorg. Med. Chem. Lett..

[95] Xiao X., Xu Y., Yu X., Chen Y., Zhao W., Xie Z., Zhu X., Xu H., Yang Y., Zhang P. (2023). Discovery of imidazo[1,2-b]pyridazine macrocyclic derivatives as novel ALK inhibitors capable of combating multiple resistant mutants. Bioorg. Med. Chem. Lett..

[96] Di Marco C.N., Terrell L., Sanchez R., Rueda L., Shuster L., Nartey E.N., McHugh C., Mack J.F., Shu A., Tian X. (2023). Design and synthesis of aminopyridine containing biaryls reducing c-MYC protein levels in cells. Bioorg. Med. Chem. Lett..

[97] Li C., Han Y., Wang Z., Yu Y., Wang C., Ren Z., Guo Y., Zhu T., Li X., Dong S. (2023). Function-oriented synthesis of Imidazo[1,2-a]pyrazine and Imidazo[1,2-b]pyridazine derivatives as potent PI3K/mTOR dual inhibitors. Eur. J. Med. Chem..

[98] Dimitrov T., Moschopoulou A.A., Seidel L., Kronenberger T., Kudolo M., Poso A., Geibel C., Wölffing P., Dauch D., Zender L. (2023). Design and Optimization of Novel Benzimidazole- and Imidazo[4,5-b]pyridine-Based ATM Kinase Inhibitors with Subnanomolar Activities. J. Med. Chem..

[99] Ren Y., Wang Y., Liu J., Liu T., Yuan L., Wu C., Yang Z., Chen J. (2023). X-ray Crystal Structure-Guided Discovery of Novel Indole Analogues as Colchicine-Binding Site Tubulin Inhibitors with Immune-Potentiating and Antitumor Effects against Melanoma. J. Med. Chem..

[100] Xiang S., Wang J., Huang H., Wang Z., Song X., Zhou Y., Jin F., He X., Zhang Z.-M., Tu Z. (2023). Switch type I to type II TRK inhibitors for combating clinical resistance induced by xDFG mutation for cancer therapy. Eur. J. Med. Chem..

[101] Wang Z., Wang J., Wang Y., Xiang S., Zhou H., Song S., Song X., Tu Z., Zhou Y., Ding K. (2023). Structure-Based Optimization of the Third Generation Type II Macrocycle TRK Inhibitors with Improved Activity against Solvent-Front, xDFG, and Gatekeeper Mutations. J. Med. Chem..

[102] Guo Y., Zou Y., Chen Y., Deng D., Zhang Z., Liu K., Tang M., Yang T., Fu S., Zhang C. (2023). Design, synthesis and biological evaluation of purine-based derivatives as novel JAK2/BRD4(BD2) dual target inhibitors. Bioorg. Chem..

[103] Cárdenas E.L., O’Rourke R.L., Menon A., Meagher J., Stuckey J., Garner A.L. (2023). Design of Cell-Permeable Inhibitors of Eukaryotic Translation Initiation Factor 4E (eIF4E) for Inhibiting Aberrant Cap-Dependent Translation in Cancer. J. Med. Chem..

[104] Kim G., Hou X., Byun W.S., Kim G., Jarhad D.B., Lee G., Hyun Y.E., Yu J., Lee C.S., Qu S. (2023). Structure–Activity Relationship of Truncated 2,8-Disubstituted-Adenosine Derivatives as Dual A2A/A3 Adenosine Receptor Antagonists and Their Cancer Immunotherapeutic Activity. J. Med. Chem..

[105] Moi D., Bonanni D., Belluti S., Linciano P., Citarella A., Franchini S., Sorbi C., Imbriano C., Pinzi L., Rastelli G. (2023). Discovery of potent pyrrolo-pyrimidine and purine HDAC inhibitors for the treatment of advanced prostate cancer. Eur. J. Med. Chem..

[106] Chen Y., Meng L., Wang W., Ye L., Huang L., Wang C., Wang S., Li M., Pei Y., Zhang S. (2023). Design, synthesis and biological evaluation of novel DCLK1 inhibitor containing purine skeleton for the treatment of pancreatic cancer. Eur. J. Med. Chem..

[107] Summers H.S., Lewis W., Williams H.E.L., Bradshaw T.D., Moody C.J., Stevens M.F.G. (2023). Discovery of new imidazotetrazinones with potential to overcome tumor resistance. Eur. J. Med. Chem..

[108] Sowmya D.V., Gari D.K., Daggupati T., Chitrala K.N., Yeguvapalli S., Adivireddy P., Padmavathi V. (2023). Synthesis, Antimicrobial, Cytotoxic and Molecular Docking Studies of Bis(azolylsulfonyl)pyrrole Dicarboxamides. Polycycl. Aromat. Compd..

[109] Hirose Y., Sato S., Hashiya K., Bando T., Sugiyama H. (2023). Anticancer Activities of DNA-Alkylating Pyrrole-Imidazole Polyamide Analogs Targeting RUNX Transcription Factors against p53-Mutated Pancreatic Cancer PANC-1 Cells. J. Med. Chem..

[110] Specker E., Wesolowski R., Schütz A., Matthes S., Mallow K., Wasinska-Kalwa M., Winkler L., Oder A., Alenina N., Pleimes D. (2023). Structure-Based Design of Xanthine-Imidazopyridines and -Imidazothiazoles as Highly Potent and In Vivo Efficacious Tryptophan Hydroxylase Inhibitors. J. Med. Chem..

[111] Syed T., Asiri Y.I., Shaheen S. (2023). Synthesis and Anticancer Assessment of Various Amide Derivatives of Imidazo[2,1-b]Oxazoles as Anticancer Agents. Polycycl. Aromat. Compd..

[112] Kuttruff C.A., Fleck M., Carotta S., Arnhof H., Bretschneider T., Dahmann G., Gremel G., Grube A., Handschuh S., Heimann A. (2023). Discovery of BI 7446: A Potent Cyclic Dinucleotide STING Agonist with Broad-Spectrum Variant Activity for the Treatment of Cancer. J. Med. Chem..

[113] Hassan A.Y., Abou-Amra E.S., El-Sebaey S.A. (2023). Design and synthesis of new series of chiral pyrimidine and purine analogs as COX-2 inhibitors: Anticancer screening, molecular modeling, and in silico studies. J. Mol. Struct..

[114] Hasanvand Z., Oghabi Bakhshaiesh T., Peytam F., Firoozpour L., Hosseinzadeh E., Motahari R., Moghimi S., Nazeri E., Toolabi M., Momeni F. (2023). Imidazo[1,2-a]quinazolines as novel, potent EGFR-TK inhibitors: Design, synthesis, bioactivity evaluation, and in silico studies. Bioorg. Chem..

[115] Husseiny E.M., Abulkhair H.S., Saleh A., Altwaijry N., Zidan R.A., Abdulrahman F.G. (2023). Molecular overlay-guided design of new CDK2 inhibitor thiazepinopurines: Synthesis, anticancer, and mechanistic investigations. Bioorg. Chem..

[116] Sadula A., Gaddhe L. (2023). Synthesis, computational studies and biological evaluation of novel Acenaphthoquinone-imidazole derivatives as dual inhibitors of HSP90 and Topo II in cancer therapy. Results Chem..

[117] Yuan J., Liu Z., Dong Y., Gao F., Xia X., Wang P., Luo Y., Zhang Z., Yan D., Zhang W. (2023). Pioneering 4,11-Dioxo-4,11-dihydro-1H-anthra[2,3-d]imidazol-3-ium Compounds as Promising Survivin Inhibitors by Targeting ILF3/NF110 for Cancer Therapy. J. Med. Chem..

[118] Nnabuike G.G., Salunke-Gawali S., Patil A.S., Butcher R.J., Obaleye J.A., Ashtekar H., Prakash B. (2023). Cobalt(II) complexes containing mefenamic acid with imidazole and pyridine based auxiliary ligands: Synthesis, structural investigation and cytotoxic evaluation. J. Mol. Struct..

[119] Pilon A., Avecilla F., Mohai M., Enyedy É.A., Rácz B., Spengler G., Garcia M.H., Valente A. (2023). First iron(II) organometallic compound acting as ABCB1 inhibitor. Eur. J. Med. Chem..

[120] Waheeb A.S. (2023). Spectroscopic, characterization and bioactivity studies of new Ni (II), Cu (II) and Ag (I) complexes with didentate (N,N) donar azo dye ligand. J. Mol. Struct..

[121] Al-Adilee K.J., Abbas Abood M. (2023). Synthesis, characterization, and cytotoxic activity of some transition metal complexes with didentate (N,N) donor azo dye ligand derived from p-Anisidine. Results Chem..

[122] Fnfoon D.Y., Al-Adilee K.J. (2023). Synthesis and spectral characterization of some metal complexes with new heterocyclic azo imidazole dye ligand and study biological activity as anticancer. J. Mol. Struct..

[123] Jawad S.H., Al-Adilee K.J. (2023). Synthesis, spectroscopic characterization and biological activities as an anticancer and antioxidant of the Pd(II) and Pt(IV) complexes with a new azo dye ligand derived from 5-methyl imidazole. J. Mol. Struct..

[124] Kumari P., Ghosh S., Acharya S., Mitra P., Roy S., Ghosh S., Maji M., Singh S., Mukherjee A. (2023). Cytotoxic Imidazolyl-Mesalazine Ester-Based Ru(II) Complexes Reduce Expression of Stemness Genes and Induce Differentiation of Oral Squamous Cell Carcinoma. J. Med. Chem..

[125] Kapitza P., Scherfler A., Salcher S., Sopper S., Cziferszky M., Wurst K., Gust R. (2023). Reaction Behavior of [1,3-Diethyl-4,5-diphenyl-1H-imidazol-2-ylidene] Containing Gold(I/III) Complexes against Ingredients of the Cell Culture Medium and the Meaning on the Potential Use for Cancer Eradication Therapy. J. Med. Chem..

[126] Zhang J.-J., Xu Q.-J., Schmidt C., Maaty M.A.A.E., Song J., Yu C., Zhou J., Han K., Sun H., Casini A. (2023). Elucidating the Multimodal Anticancer Mechanism of an Organometallic Terpyridine Platinum(II) N-Heterocyclic Carbene Complex against Triple-Negative Breast Cancer In Vitro and In Vivo. J. Med. Chem..

[127] Yang Z., Bian M., Lv L., Chang X., Wen Z., Li F., Lu Y., Liu W. (2023). Tumor-Targeting NHC–Au(I) Complex Induces Immunogenic Cell Death in Hepatocellular Carcinoma. J. Med. Chem..

[128] Patagar D.N., Kusanur R., Batakurki S.R., Patra S.M., Patil N.R., Patil J.H. (2023). 8-benzimidazolyl coumarin-3-oxadiazoles—Synthesis, docking studies and Anti-proliferative evaluation against breast cancer. J. Mol. Struct..

[129] Wang Y., Liu Y., Ge T., Tang J., Wang S., Gao Z., Chen J., Xu J., Gong P., Zhao Y. (2023). Based on 2-(difluoromethyl)-1-[4,6-di(4-morpholinyl)-1,3,5-triazin-2-yl]-1H-benzimidazole (ZSTK474), design, synthesis and biological evaluation of novel PI3Kα selective inhibitors. Bioorg. Chem..

[130] Wang Y., Nie G., Wang X., Ge W., Zhang Y. (2023). Development of small molecule inhibitors targeting RNA helicase DHX33 as anti-cancer agents. Bioorg. Med. Chem. Lett..

[131] Fang Y., Tan Q., Zhou H., Xu J., Gu Q. (2023). Discovery and optimization of 2-(trifluoromethyl)benzimidazole derivatives as novel ferroptosis inducers in vitro and in vivo. Eur. J. Med. Chem..

[132] Coşkun G.P., Sahin Z., Erdoğan Ö., Çevik Ö., Biltekin S.N., Yurttas L., Berk B., Ülgen M., Demirayak Ş. (2023). Discovery of novel potent human chondrosarcoma (SW1353) inhibitors: 4-(2/3/4-pyridyl)thiazole 2-acetamide derivatives. J. Mol. Struct..

[133] Duan Y., Cheng H., Zhuang L., Xia J., Xu Y., Zhang R., Sun R., Lu T., Chen Y. (2023). Discovery of Thieno [3,2-d]pyrimidine derivatives as potent and selective inhibitors of ataxia telangiectasia mutated and Rad3 related (ATR) kinase. Eur. J. Med. Chem..

[134] Radwan M.O., Toma T., Arakaki Y., Kamo M., Inoue N., Koga R., Otsuka M., Tateishi H., Fujita M. (2023). New insight into the bioactivity of substituted benzimidazole derivatives: Repurposing from anti-HIV activity to cell migration inhibition targeting hnRNP M. Biorg. Med. Chem..

[135] Fan Y., Luo F., Su M., Li Q., Zhong T., Xiong L., Li M., Yuan M., Wang D. (2023). Structure optimization, synthesis, and biological evaluation of 6-(2-amino-1H-benzo[d]imidazole-6-yl)-quinazolin-4(3H)-one derivatives as potential multi-targeted anticancer agents via Aurora A/PI3K/BRD4 inhibition. Bioorg. Chem..

[136] Kadam P.R., Bodke Y.D., Pushpavathi I., Satyanarayan N.D., Nippu B.N. (2023). Synthesis, characterization, DFT and biological study of new methylene thio-linked coumarin derivatives. J. Mol. Struct..

[137] Fu S., Liu J., Li C., Wei J., Yue H., Yang A., Wang K., Wu Y., Hou Y., Zhao Y. (2023). Structure-based drug design, synthesis, and biological evaluation of novel 1,3,5-triazine or pyrimidine derivatives containing benzoyl hydrazine moiety as PI3Kα selective inhibitors. Bioorg. Chem..

[138] Li Y., Liu Y., Zhang D., Chen J., Yang G., Tang P., Yang C., Liu J., Zhang J., Ouyang L. (2023). Discovery, Synthesis, and Evaluation of Novel Dual Inhibitors of a Vascular Endothelial Growth Factor Receptor and Poly(ADP-Ribose) Polymerase for BRCA Wild-Type Breast Cancer Therapy. J. Med. Chem..

[139] Liu L., Zhang L., Chen X., Yang K., Cui H., Qian R., Zhao S., Wang L., Su X., Zhao M. (2023). Design and synthesis of 1H-benzo[d]imidazole selective HDAC6 inhibitors with potential therapy for multiple myeloma. Eur. J. Med. Chem..

[140] Zhang L., Zhen Y., Feng L., Li Z., Lu Y., Wang G., Ouyang L. (2023). Discovery of a novel dual-target inhibitor of CDK12 and PARP1 that induces synthetic lethality for treatment of triple-negative breast cancer. Eur. J. Med. Chem..

[141] Abdullah M.i.N., Abd Hamid S., Muhamad Salhimi S., Jalil N.A.S., Al-Amin M., Jumali N.S. (2023). Design and synthesis of 1-sec/tert-butyl-2-chloro/nitrophenylbenzimidazole derivatives: Molecular docking and in vitro evaluation against MDA-MB-231 and MCF-7 cell lines. J. Mol. Struct..

[142] Chen N.-N., Zhang H., Zhu Q.-S., Zeng T., Dai W., Zhou Y.-L., Xin G.-F., Wu B.-D., Gong S.-J., Jiang Z.-Y. (2023). Development of Orally Bioavailable Amidobenzimidazole Analogues Targeting Stimulator of Interferon Gene (STING) Receptor. J. Med. Chem..

[143] Liu X., Wang M., Yang M., Sun H., Wang B., Pan X., Chen X., Jin J., Wang X. (2023). Structure-activity relationship study of amidobenzimidazole derivatives as stimulator of interferon genes (STING) agonists. Eur. J. Med. Chem..

[144] Modukuri R.K., Monsivais D., Li F., Palaniappan M., Bohren K.M., Tan Z., Ku A.F., Wang Y., Madasu C., Li J.-Y. (2023). Discovery of Highly Potent and BMPR2-Selective Kinase Inhibitors Using DNA-Encoded Chemical Library Screening. J. Med. Chem..

[145] Wang R., Du T.-T., Liu W.-Q., Liu Y.-C., Yang Y.-D., Hu J.-P., Ji M., Yang B.-B., Li L., Chen X.-G. (2023). Discovery, Optimization, and Evaluation of Novel N-(Benzimidazol-5-yl)-1,3,4-thiadiazol-2-amine Analogues as Potent STAT3 Inhibitors for Cancer Treatment. J. Med. Chem..

[146] Jeon M.J., Lee H., Jo S., Kang M., Jeong J.H., Jeong S.H., Lee J.-Y., Song G.Y., Choo H., Lee S. (2023). Discovery of novel amidobenzimidazole derivatives as orally available small molecule modulators of stimulator of interferon genes for cancer immunotherapy. Eur. J. Med. Chem..

[147] Ko B., Jang Y., Kim M.H., Lam T.T., Seo H.K., Jeong P., Choi M., Kang K.W., Lee S.-D., Park J.-H. (2023). Discovery of benzimidazole-indazole derivatives as potent FLT3-tyrosine kinase domain mutant kinase inhibitors for acute myeloid leukemia. Eur. J. Med. Chem..

[148] Bradley E., Fusani L., Chung C.-W., Craggs P.D., Demont E.H., Humphreys P.G., Mitchell D.J., Phillipou A., Rioja I., Shah R.R. (2023). Structure-Guided Design of a Domain-Selective Bromodomain and Extra Terminal N-Terminal Bromodomain Chemical Probe. J. Med. Chem..

[149] Cui B., Wang Y., Zhao Z., Fan L., Jiao Y., Li H., Feng J., Tang W., Lu T., Chen Y. (2023). Discovery of 3-(1H-benzo[d]imidazole-2-yl)-1H-pyrazol-4 -amine derivatives as novel and potent syk inhibitors for the treatment of hematological malignancies. Eur. J. Med. Chem..

[150] Theodore C.E., Sivaiah G., Prasad S.B.B., Kumar K.Y., Raghu M.S., Alharethy F., Prashanth M.K., Jeon B.-H. (2023). Design, synthesis, anticancer activity and molecular docking of novel 1H-benzo[d]imidazole derivatives as potential EGFR inhibitors. J. Mol. Struct..

[151] Guo S., Jia T., Xu X., Yang F., Xiao S., Hou Z., Xu H., Ma S., Liu X., Luo C. (2023). Design, synthesis of novel benzimidazole derivatives as ENL inhibitors suppressing leukemia cells viability via downregulating the expression of MYC. Eur. J. Med. Chem..

[152] Shinde R.B., Pansare D.N., Shelke R.N., Sarkate A.P., Tiwari S.V., Bangal M.N., Bhagat D.S., Zine A.M. (2023). A facile synthesis and characterization of some novel benzimidazole derivatives. Results Chem..

[153] Ahmed Saleh Alzahrani S., Nazreen S., Elhenawy A.A., Neamatallah T., Mahboob M. (2023). Synthesis, Biological Evaluation, and Molecular Docking of New Benzimidazole-1,2,3-Triazole Hybrids as Antibacterial and Antitumor Agents. Polycycl. Aromat. Compd..

[154] Singh S., Paul S., Brás N.F., Kundu C.N., Karthikeyan C., Moorthy N.S.H.N. (2023). Design, synthesis, and anticancer activity of some novel 1H-benzo[d]imidazole-5-carboxamide derivatives as fatty acid synthase inhibitors. Bioorg. Chem..

[155] Bondock S., Albormani O., Fouda A.M. (2023). Expedient Synthesis and Antitumor Evaluation of Novel Azaheterocycles from Thiazolylenaminone. Polycycl. Aromat. Compd..

[156] Zhang S., Qing L., Wang Z., Zhang Y., Li Y., Fang H., Liu Y., He H. (2023). Design and Structural Optimization of Methionine Adenosyltransferase 2A (MAT2A) Inhibitors with High In Vivo Potency and Oral Bioavailability. J. Med. Chem..

[157] Rao G.V.N., Taneja A.K., Tej M.B., Sri K.N., Vijayavardhini S., Dandamudi S., Chinnamaneni S.V., Kapavarapu R., Rao M.V.B., Pal M. (2023). Sonochemical synthesis, docking studies and in vitro evaluation of imidazo-1,3-oxazinone derivatives as potential inhibitors of TNF-α. J. Mol. Struct..

[158] Horai Y., Suda N., Uchihashi S., Katakuse M., Shigeno T., Hirano T., Takahara J., Fujita T., Mukoyama Y., Haga Y. (2023). Discovery of a potent, orally available tricyclic derivative as a novel BRD4 inhibitor for melanoma. Bioorg. Med. Chem..

[159] Hassan A., Ashraf R., Iqbal M.A., El-Naggar M., Tahira S.A., Hayat K. (2024). Synthesis, characterization and molecular docking of benz-imidazolium Se-adducts: Antimicrobial and anticancer studies. J. Mol. Struct..

[160] Thankan R.S., Thomas E., Purushottamachar P., Weber D.J., Njar V.C.O. (2023). Salinization Dramatically Enhance the Anti-Prostate Cancer Efficacies of AR/AR-V7 and Mnk1/2 Molecular Glue Degraders, Galeterone and VNPP433-3β Which Outperform Docetaxel and Enzalutamide in CRPC CWR22Rv1 Xenograft Mouse Model. Bioorg. Chem..

[161] Jackson J.J., Siegmund A.C., Bai W.-J., Reed A.B., Birkholz A.B., Campuzano I.D.G., Créquer-Grandhomme A., Hu R., Modak R.V., Sudom A. (2023). Imidazolone as an Amide Bioisostere in the Development of β-1,3-N-Acetylglucosaminyltransferase 2 (B3GNT2) Inhibitors. J. Med. Chem..

[162] Choi J.Y., Noguchi Y., Alburger J.M., Bayle S., Chung E., Grant W., Chaikuad A., Knapp S., Duckett D.R., Roush W.R. (2023). Structure-Based Development of Isoform-Selective Inhibitors of Casein Kinase 1ε vs Casein Kinase 1δ. J. Med. Chem..

[163] Chen Y., Li W., Yang Y., Zhong R., Hu H., Huang C., Chen J., Liang L., Liu Y. (2023). Significant increase of anticancer efficacy in vitro and in vivo of liposome entrapped ruthenium(II) polypyridyl complexes. Eur. J. Med. Chem..

[164] Yuan Y., Zhang Y., Chen J., Huang C., Liu H., Li W., Liang L., Wang Y., Liu Y. (2023). Synthesis, biological evaluation of novel iridium(III) complexes targeting mitochondria toward melanoma B16 cells. Eur. J. Med. Chem..

[165] Hou M., Li H.C., An N., Pang S.Y., Li W.G., Tong J. (2023). Synthesis, crystal structures and anticancer studies of Ni (II), Co (III) and Zn (II) complexes based on 5-bromosalicylaldehyde-2-(2-aminophenyl)benzimidazole Schiff base. J. Mol. Struct..

